# Genetic associations of plasma proteomics with dementia subtypes and neuroimaging markers

**DOI:** 10.1002/dad2.70202

**Published:** 2025-10-21

**Authors:** Ahmed M. Salih, Janek Salatzki, Yuhe Wang, Tesfamariam Akilu, Cynthia Maldonado, Masud Husain, Stefan Neubauer, Anya Topiwala, André Altmann, Zahra Raisi‐Estabragh

**Affiliations:** ^1^ William Harvey Research Institute National Institute for Health Research Barts Biomedical Research Centre Queen Mary University of London London UK; ^2^ Department of Population Health Sciences University of Leicester Leicester UK; ^3^ PRIME Lab Scientific Research Centre University of Zakho Zakho Iraq; ^4^ Department of Cardiology Angiology and Pneumology Heidelberg University Hospital Heidelberg Germany; ^5^ Diabetes Research Centre University of Leicester Leicester UK; ^6^ National Institute for Health Research Leicester Biomedical Research Centre University of Leicester and University Hospitals of Leicester NHS Trust Leicester UK; ^7^ Department of Cardiology, College of Health Sciences Addis Ababa University Addis Ababa Ethiopia; ^8^ Wellcome Centre for Integrative Neuroimaging University of Oxford Oxford UK; ^9^ Department of Experimental Psychology University of Oxford Oxford UK; ^10^ Nuffield Department of Clinical Neurosciences University of Oxford John Radcliffe Hospital Oxford UK; ^11^ Division of Cardiovascular Medicine, Radcliffe Department of Medicine National Institute for Health Research Oxford Biomedical Research Centre University of Oxford Oxford University Hospitals NHS Foundation Trust Oxford UK; ^12^ Nuffield Department of Population Health, Big Data Institute University of Oxford, Warneford Hospital Oxford UK; ^13^ Department of Medical Physics and Biomedical Engineering The UCL Hawkes Institute University College London London UK; ^14^ Barts Heart Centre St. Bartholomew's Hospital Barts Health NHS Trust London UK

**Keywords:** Alzheimer's disease, dementia, genome‐wide association study, neuroimaging, Parkinson's disease dementia, proteomics, vascular dementia

## Abstract

**INTRODUCTION:**

Dementia is a rising global health challenge. Advances in large‐scale proteomics and genetic databases have enabled high‐throughput screening approaches to uncover novel mechanistic pathways and therapeutic targets.

**METHODS:**

This study used a Mendelian randomization framework to examine genetic associations of 2172 plasma proteins (UK Biobank, *n* = 54,219) with: (1) dementia subtypes (FinnGen, *n* = 429,209), including Alzheimer's disease (*n* = 12,348), vascular dementia (*n* = 2667), and Parkinson's disease dementia (*n* = 589); and (2) global neuroimaging markers (UK Biobank), including white matter hyperintensities (*n* = 42,310), fractional anisotropy (*n* = 17,663), and mean diffusivity (*n* = 17,467).

**RESULT:**

Multiple potential causal protein–outcome relationships were identified, corroborating known associations (e.g., apolipoprotein E, synaptosomal‐associated protein 25) and uncovering more novel proteins (e.g., butyrophilin subfamily 3 member A2, granzyme A, contactin‐2, and trefoil factor 3) potentially involved in dementia disease processes.

**DISCUSSION:**

The identified proteins have diverse functions spanning immune regulation, cellular proliferation, neuronal stability, and neuroinflammation. The findings increase our understanding of disease processes governing cognitive health and highlight candidate proteins with potential as new disease biomarkers or therapeutic targets.

**Highlights:**

- We used Mendelian randomization to link 2172 plasma proteins to dementia and brain imaging traits.
- Apolipoprotein E, triggering receptor expressed on myeloid cells 2, and Fc receptor‐like 3 showed protective associations across dementia subtypes.
- Butyrophilin subfamily 3 member A2, granzyme A, contactin‐2, and trefoil factor 3 were uncovered as novel dementia‐associated proteins.
- Immune, metabolic, and vascular pathways were implicated in the etiology of dementia.

## 1 BACKGROUND

Dementia encompasses a spectrum of neurodegenerative disorders characterized by cognitive decline, memory impairment, and loss of functional independence, affecting > 55 million individuals worldwide—a number projected to triple by 2050.^1^ Alzheimer's disease (AD) is the most prevalent subtype, accounting for 60% to 70% of cases, followed by vascular dementia (VD), and Parkinson's disease dementia (PDD).^2^, ^3^ These conditions arise from complex pathological processes, including protein aggregation, synaptic dysfunction, neuronal damage, vascular dysfunction, and neuroinflammation. Despite advances in understanding the pathophysiology of dementia, the precise mechanisms driving disease progression remain incompletely understood, highlighting the need for innovative approaches to uncover causative pathways and therapeutic targets.

Plasma proteins play a pivotal role in the pathogenesis of dementia, serving as biomarkers, prognostic indicators, and therapeutic targets.^4^ Proteomic analyses have demonstrated potential for advancing early diagnosis and risk stratification of dementia.^5^ However, observational studies exploring protein–disease associations are limited by confounding and reverse causation, hindering the establishment of causal relationships.^5^, ^6^ To address these challenges, integrating genetic data with proteomics offers a powerful framework for assessing the roles of proteins in the etiology of dementia.^7^

The recent emergence of large‐scale biomedical databases that include both proteomics and genetics profiling has enabled genome wide association studies (GWASs), providing new insights into the genetic determinants of individual proteomic markers. These genetic variants can be used as instrumental variables (IVs) within Mendelian randomization (MR) frameworks to examine the association of genetically predicted protein levels with dementia outcomes to enhance mechanistic understanding, while minimizing biases from traditional observational methods.^8^ Previous MR studies have demonstrated individual protein–dementia relationships, such as links between amyloid beta (Aβ) and tau pathology proteins with AD.^9^ The availability of large‐scale proteomics and genetic datasets enables implementation of high‐throughput screening methods to identify novel mechanistic pathways, disease biomarkers, and therapeutic targets.^10^

This study used a MR framework to examine genetic associations of an extensive panel of plasma proteomics with (1) dementia subtypes and (2) neuroimaging markers. The work represents the most comprehensive evaluation of associations between proteomic markers and dementia to date, providing new insights into mechanistic pathways and potential therapeutic targets.

## 2 METHODS

### 2.1 Study design

**FIGURE 1:**
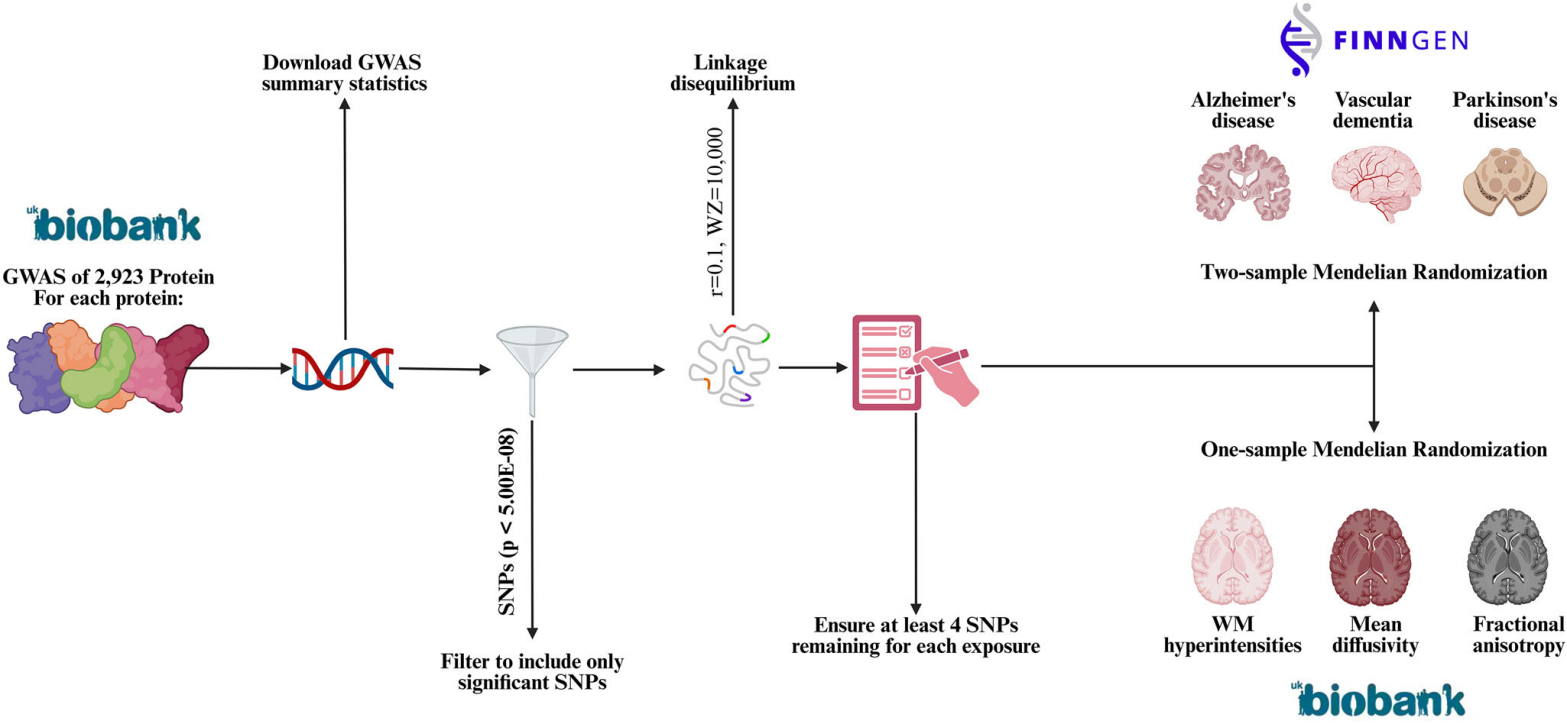
Overview of the study methods. Number of analyses for each of AD, VD, and PDD: 2172, number of analyses for each of WMH, FA, and MD: 2169. AD, Alzheimer's disease; FD, fractional anisotropy; FDR, false discovery rate; GWAS, genome‐wide association study; IVW, inverse variance weighted; MD, mean diffusivity; MR, Mendelian randomization; PDD, Parkinson's disease dementia; SNPs, single‐nucleotide polymorphisms; VD, vascular dementia; WMH, white matter hyperintensity.

A two‐sample MR framework was applied to evaluate relationships between plasma proteins and dementia subtypes (Figure 1). Relevant GWASs were identified through a systematic literature search, covering plasma proteins (exposures), three dementia subtypes (outcomes), and three neuroimaging markers (outcomes). Genetic variants—single‐nucleotide polymorphisms (SNPs), significantly associated with plasma proteins were selected as IVs based on established thresholds (*P* < 5 × 10^−^⁸) and linkage disequilibrium (LD) criteria. The study adhered to the Strengthening the Reporting of Observational Studies in Epidemiology using Mendelian randomization (STROBE‐MR) guidelines, ensuring methodological robustness and transparency.^11^

### 2.2 Data sources

The largest available GWAS of plasma proteomics was selected to identify the exposure IVs. In their analysis of 2923 plasma proteins from 54,219 UK Biobank participants, Sun et al.^12^ identified 14,287 significant genetic associations with plasma proteins, including both *cis*‐ and *trans*‐protein quantitative trait loci (pQTLs).

### 2.3 Outcome

The outcome dataset for dementia subtypes was sourced from the FinnGen GWAS database.^13^, ^14^ This dataset includes genetic and health record data from > 429,209 participants of Finnish ancestry. FinnGen, an ongoing project initiated in 2017, integrates genomic data with electronic health records to facilitate comprehensive exploration of the human genome and its links to various diseases. The outcomes of interest included three dementia subtypes: VD, AD, and PDD. Detailed information on sample size, case proportions, and population characteristics is provided in Table S1 in supporting information.

**RESEARCH IN CONTEXT**

1. **Systematic review**: We conducted a targeted literature review to inform protein selection and interpretation. We then performed structured searches to evaluate the evidence for their involvement in neurodegeneration. While some prior Mendelian randomization (MR) studies explored specific proteins such as apolipoprotein E (apoE) and tau, no large‐scale MR analyses had assessed plasma proteomics across dementia subtypes and neuroimaging markers.
2. **Interpretation**: This study presents the most comprehensive MR analysis to date examining 2172 genetically predicted plasma proteins in relation to Alzheimer's disease, vascular dementia, Parkinson's disease dementia, and neuroimaging biomarkers. We confirm known associations (e.g., apoE, triggering receptor expressed on myeloid cells 2) and identify novel candidates (e.g., butyrophilin subfamily 3 member A2, Fc receptor‐like 3, and protein MENT), implicating key immune, inflammatory, and metabolic pathways.
3. **Future directions**: Further studies should validate findings in diverse populations and assess the mechanistic roles and biomarker potential of newly identified proteins.

### 2.4 Neuroimaging markers

Brain magnetic resonance imaging (MRI) marker datasets were sourced from the UK Biobank GWAS by Persyn et al.,^15^ and included global white matter hyperintensities (WMHs), fractional anisotropy (FA), and mean diffusivity (MD). These markers were selected because they reflect global white matter micro‐injuries (WMHs), brain connectivity (FA), and white matter microstructural integrity (MD) and have established links to cognitive impairment and progression to dementia.^16^, ^17^

Total WMH volumes were derived from T1‐weighted and T2 fluid‐attenuated inversion recovery (FLAIR) images. FA and MD values, using diffusion tensor imaging and diffusion MRI, and calculated for 48 specific white matter tracts. FA measures the directional coherence of water diffusion in brain tissue, providing insights into white matter integrity, while MD quantifies the overall diffusivity of water molecules, reflecting tissue microstructure.

Principal component (PC) analysis was applied to FA and MD values across the different tracts, and the first PC was used to run the GWASs. Quality control was applied at SNP (e.g., minor allele frequency) and individual level (e.g., kinship coefficient). The final GWAS analysis included ≈ 9.7 million SNPs.^15^ Detailed information on sample size and case proportions for brain imaging traits in the GWAS is provided in Table S2 in supporting information.

### 2.5 Statistical analysis

Figure 1 shows an overview of the analysis, including the selection of IVs and the implementation of MR. For each protein, GWAS summary statistics were obtained and filtered to include only significant SNPs based on the standard GWAS *P* value threshold (*P* < 5 × 10^−^⁸). To ensure independence among variants, LD pruning with standard settings was applied using an LD threshold of *r* ^2^ = 0.1, a window size of 10,000 base pairs, and reference data from a European population. Harmonization of alleles was conducted to associate effect and reference alleles across the exposure and outcome GWAS datasets, ensuring consistency in SNP interpretation. The resulting list of independent and harmonized SNPs for each protein was then used to extract the corresponding GWAS summary statistics for the outcomes.

### 2.6 MR analysis

A two‐sample MR analysis was adopted to assess the association between plasma proteomics and the three types of dementia, as there is no overlap in the exposures and the outcome GWAS samples. A one‐sample MR analysis was used to evaluate the association between plasma proteomics and the three brain MRI biomarkers, as both the exposure and outcome samples originated from the UK Biobank. MR was implemented using an inverse‐variance weighted (IVW) method as the main analysis. To ensure the validity of the SNPs and address potential heterogeneity and directional pleiotropy, complementary analyses were conducted, including MR‐Egger regression, weighted median, and weighted mode methods.^18^ Leave‐one‐SNP‐out analyses and MR‐Egger intercept were implemented to test horizontal pleiotropy. MR pleiotropy residual sum and outlier (MR‐PRESSO) was adopting to detect and correct horizontal pleiotropic outliers.^18^ The analysis was conducted using the R package TwoSampleMR.^19^ The *P* values of IVW were corrected for multiple testing using the Bonferroni method for each outcome across all proteins. The association was considered significant if the corrected *P* value was < 0.05 in the IVW analysis and *P* value was < 0.05 in the complementary analyses. The MR analysis was conducted when at least four SNPs are included in the exposure and in the outcome because this is the minimum number of SNPs required by MR‐PRESSO to detect and correct for outliers.

### 2.7 Ethical approval

The study used publicly available GWAS summary statistics; therefore, ethical approval was not required.

## 3 RESULTS

Among the 2923 plasma proteins, 2172 were included in the analysis of dementia outcomes, and 2169 were considered for brain MRI metrics outcomes. This selection was due to the absence of valid IVs for some proteins, an insufficient number of IVs for certain analyses, or the unavailability of IVs in the GWAS outcome data. The number of SNPs passing all quality control steps and included in the MR analysis ranged from 4 to 380, depending on the protein–outcome pairing.

### 3.1 Dementia subtypes

**FIGURE 2:**
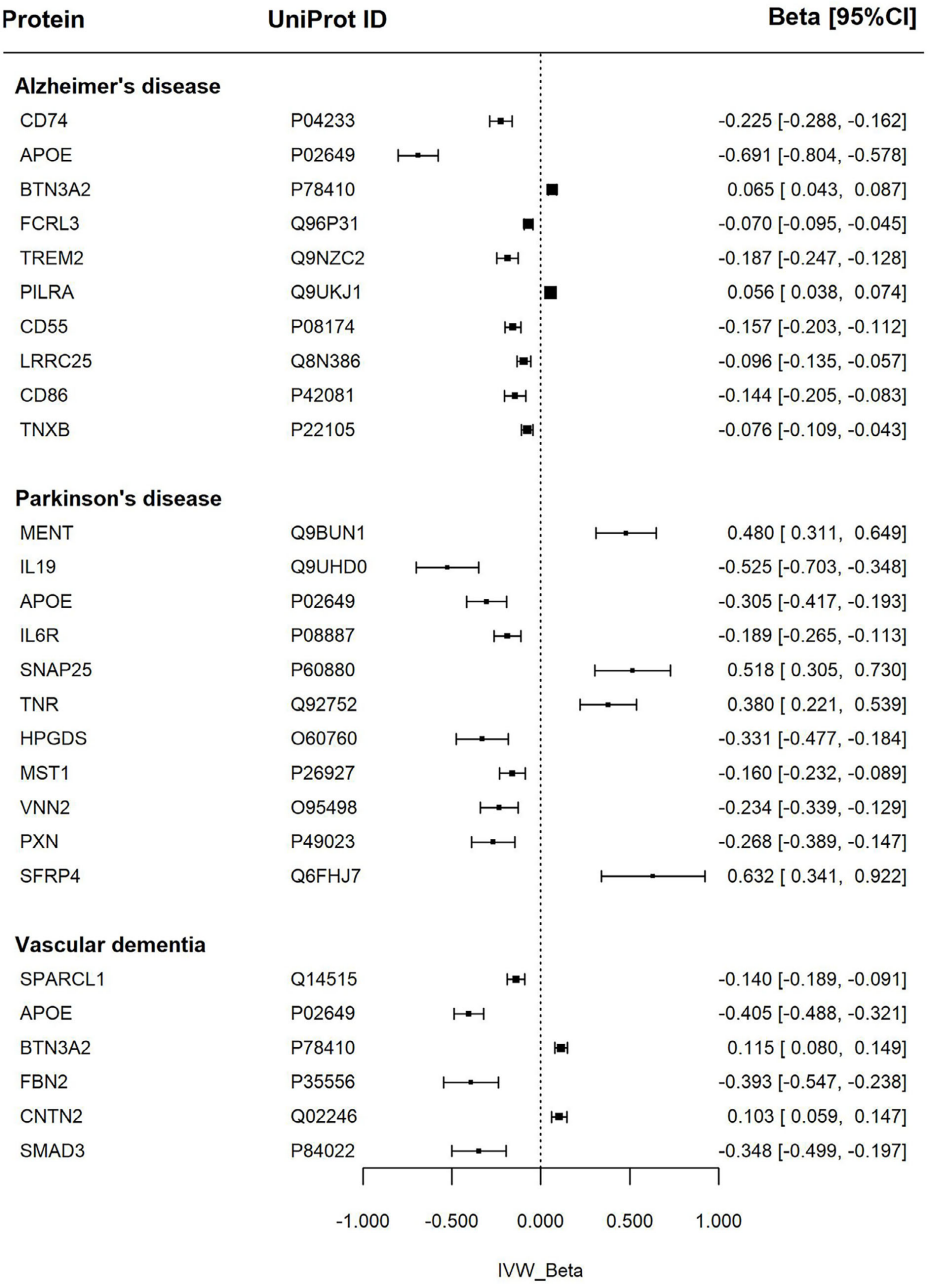
Association between proteins and dementia subtypes. Forest plot of Mendelian randomization estimates for dementia subtypes (Alzheimer's disease and vascular dementia) and Parkinson's disease in relation to specific proteins. The IVW *β* represents the inverse variance weighted effect size with corresponding 95% confidence intervals (*β* [95% CI]). apoE, apolipoprotein E; BTN3A2, butyrophilin subfamily 3 member A2; CD55, complement decay‐accelerating factor; CD74, HLA class II histocompatibility antigen gamma chain; CD86, T‐lymphocyte activation antigen; CI, confidence interval; CNTN2, contactin‐2; FBN2, fibrillin‐2; FCRL3, Fc receptor‐like protein 3; HPGDS, hematopoietic prostaglandin D synthase; IL19, interleukin‐19; IL6R, interleukin‐6 receptor subunit alpha; IVW, inverse variance weighted; LRRC25, leucine‐rich repeat‐containing protein 25; MENT, protein MENT; MST1, hepatocyte growth factor‐like protein; PILRA, paired immunoglobulin‐like type 2 receptor alpha; PXN, paxillin; SFRP4, secreted frizzled‐related protein 4; SMAD3, mothers against decapentaplegic homolog 3; SNAP‐25, synaptosomal‐associated protein 25; SPARCL1, SPARC‐like protein 1; TNR, tenascin‐R; TNXB, tenascin‐X; TREM2, triggering receptor expressed on myeloid cells 2; VNN2, pantetheine hydrolase VNN2.

After Bonferroni adjustment and complementary analyses—including MR‐Egger regression, weighted median, and weighted mode methods—the number of remaining significant associations was 10 proteins for AD, 6 for VD, and 11 for PDD (Figure 2, and Figure S1 and Tables S3 and S4 in supporting information).

### 3.2 AD

The largest effect size (by a notable distance) was the association of higher apolipoprotein E (apoE), levels with a significantly lower risk of AD. Higher CD74 molecule (CD74) and triggering receptor expressed on myeloid cells 2 (TREM2) levels were also related to lower AD risk—with second and third largest effect sizes, respectively. There were smaller magnitude negative associations with Fc receptor‐like 3 (FCRL3). Conversely, higher butyrophilin subfamily 3 member A2 (BTN3A2) levels were linked to increased risk of AD. These relationships remained robust to multiple sensitivity checks including all standard complementary analyses, multiple testing correction, and correction for pleiotropy using MR‐PRESSO, with consistent beta values and significance levels.

The remaining proteins demonstrated significant associations with AD, but with weaker supporting evidence. These comprised association of higher paired immunoglobulin‐like type 2 receptor alpha (PILRA) levels with an increased risk of AD, and small negative associations with complement decay‐accelerating factor (CD55), cluster of differentiation 86 (CD86), tenascin‐X (TNXB), and leucine‐rich repeat‐containing 25 (LRRC25).

### 3.3 PDD

The most robust results consistent across all complementary and sensitivity analyses were associations of higher apoE and interleukin‐6 receptor subunit alpha (IL6R) levels with lower risk of PDD.

Higher secreted frizzled‐related protein 4 (SFRP4), protein MENT (MENT), synaptosomal‐associated protein 25 (SNAP‐25), and tenascin‐R (TNR) levels were linked to increased risk of PDD. While higher hematopoietic prostaglandin D synthase (HPGDS), hepatocyte growth factor‐like protein (MST), pantetheine hydrolase VNN2 (VNN2), and paxillin (PXN) were linked to lower risk of PDD.

### 3.4 VD

Higher serum apoE levels were linked to significantly lower VD risk, while higher BTN3A2 levels were linked to an increased risk. These associations were robust across all complementary and sensitivity analyses.

Higher contactin‐2 (CNTN2) levels were linked to increased risk of VD. Higher SMAD family member 3 (SMAD3) and fibrillin‐2 (FBN2) levels associated with lower risk of VD.

### 3.5 Neuroimaging biomarkers

**FIGURE 3:**
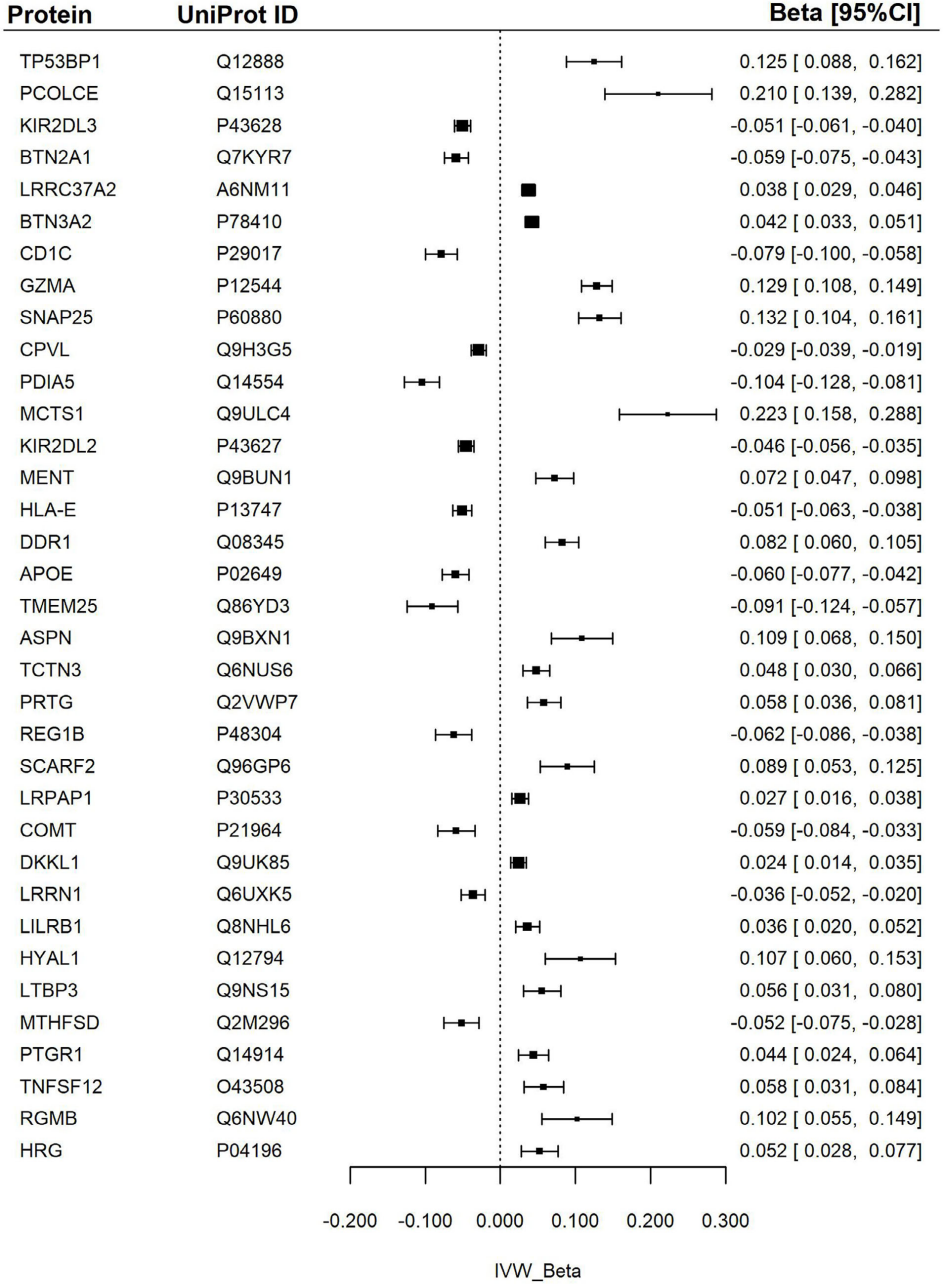
Association between proteins and white matter hyperintensity. Forest plot of Mendelian randomization estimates for white matter hyperintensities in relation to specific proteins. The IVW *β* represents the inverse variance weighted effect size with corresponding 95% confidence intervals (*β* [95% CI]). ALPP, alkaline phosphatase, placental; apoE, apolipoprotein E; APSN, asporin; BTN2A1, butyrophilin subfamily 2 member A1; BTN3A2, butyrophilin subfamily 3 member A2; CD1C, T‐cell surface glycoprotein CD1c; COMT, catechol O‐methyltransferase; CPVL, probable serine carboxypeptidase; DDR1, epithelial discoid in domain‐containing receptor 1; DKKL1, Dickkopf‐like protein 1; GZMA, granzyme A; HLA‐E, HLA class I histocompatibility antigen, alpha chain E; HRG, histidine‐rich glycoprotein; HYAL1, hyaluronidase‐1; KIR2DL2, killer cell immunoglobulin‐like receptor 2DL2; KIR2DL3, killer cell immunoglobulin‐like receptor 2DL3; LILRB1, leukocyte immunoglobulin‐like receptor subfamily B member 1; LRPAP1, Alpha‐2‐macroglobulin receptor‐associated protein; LRRC37A2, leucine‐rich repeat‐containing protein 37A2; LRRN1, leucine‐rich repeat neuronal protein 1; LTBP3; latent‐transforming growth factor beta‐binding protein 3; MCTS1, malignant T‐cell‐amplified sequence 1; MENT, protein MENT; MTHFSD, methenytetrahydrofolate synthase domain‐containing protein; PDIA5, protein disulfide‐isomerase A5; PCOLCE, procollagen C‐endopeptidase enhancer 1; PRTG, protogenin; PTGR1, prostaglandin reductase 1; REG1B, lithostathine‐1‐beta; RGMB, repulsive guidance molecule B; SCARF2, scavenger receptor class F member 2; SNAP‐25, synaptosomal‐associated protein 25; TCTN3, tectonic‐3; TMEN25, transmembrane protein 25; TNFSF12, tumor necrosis factor ligand superfamily member 12; TP53BP1, TP53‐binding protein 1.

**FIGURE 4:**
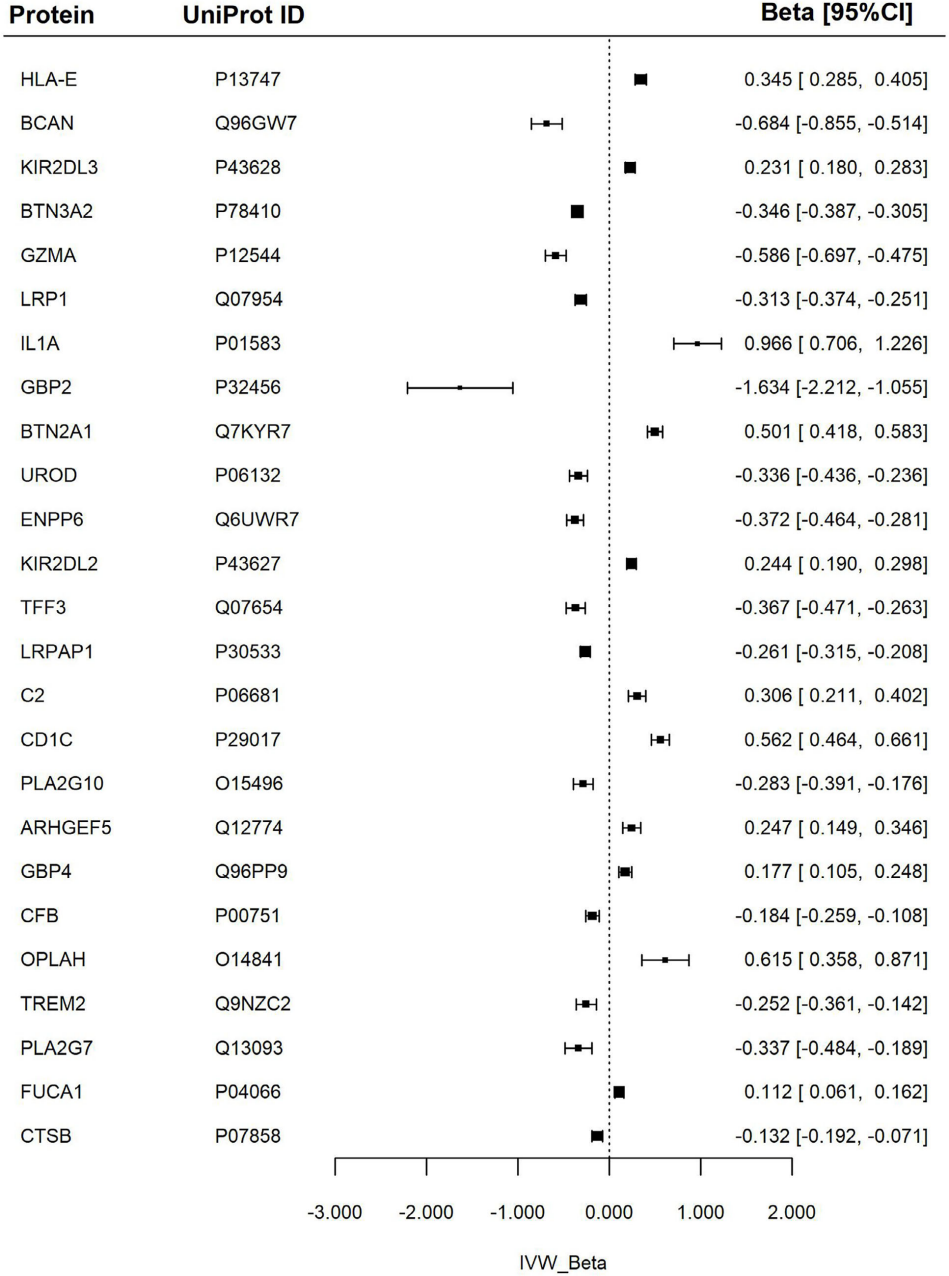
Association between proteins and fractional anisotropy. Forest plot of Mendelian randomization estimates for fractional anisotropy in relation to specific proteins. The IVW *β* represents the inverse variance weighted effect size, with corresponding 95% confidence intervals (*β* [95% CI]). ARHGEF5, rho guanine nucleotide exchange factor 5; BCAN, brevican core protein; BTN2A1, butyrophilin subfamily 2 member A1; BTN3A2, butyrophilin subfamily 3 member A2; C2, complement component 2; CFB, complement factor B; CD1C, T‐cell surface glycoprotein CD1c; CTSB, cathepsin B; ENPP6, glycerophosphocholine choline phosphodiesterase ENPP6; FUCA1, Tissue alpha‐L‐fucosidase; GBP2, Gguanylate‐binding protein 2; GBP4, guanylate‐binding protein 4; GZMA, granzyme A; HLA‐E, HLA class I histocompatibility antigen, alpha chain E; IL1A, interleukin‐1 alpha; KIR2DL2, killer cell immunoglobulin‐like receptor 2DL2; KIR2DL3, killer cell immunoglobulin‐like receptor 2DL3L; LRP1, pro‐low‐density lipoprotein receptor‐related protein 1; LRPAP1, alpha‐2‐macroglobulin receptor‐associated protein; OPLAH, 5‐oxoprolinase; PLA2G10, group 10 secretory phospholipase A2; PLA2G7, platelet‐activating factor acetylhydrolase; TFF3, trefoil factor 3; TREM2, triggering receptor expressed on myeloid cells 2; UROD, uroporphyrinogen decarboxylase.

**FIGURE 5:**
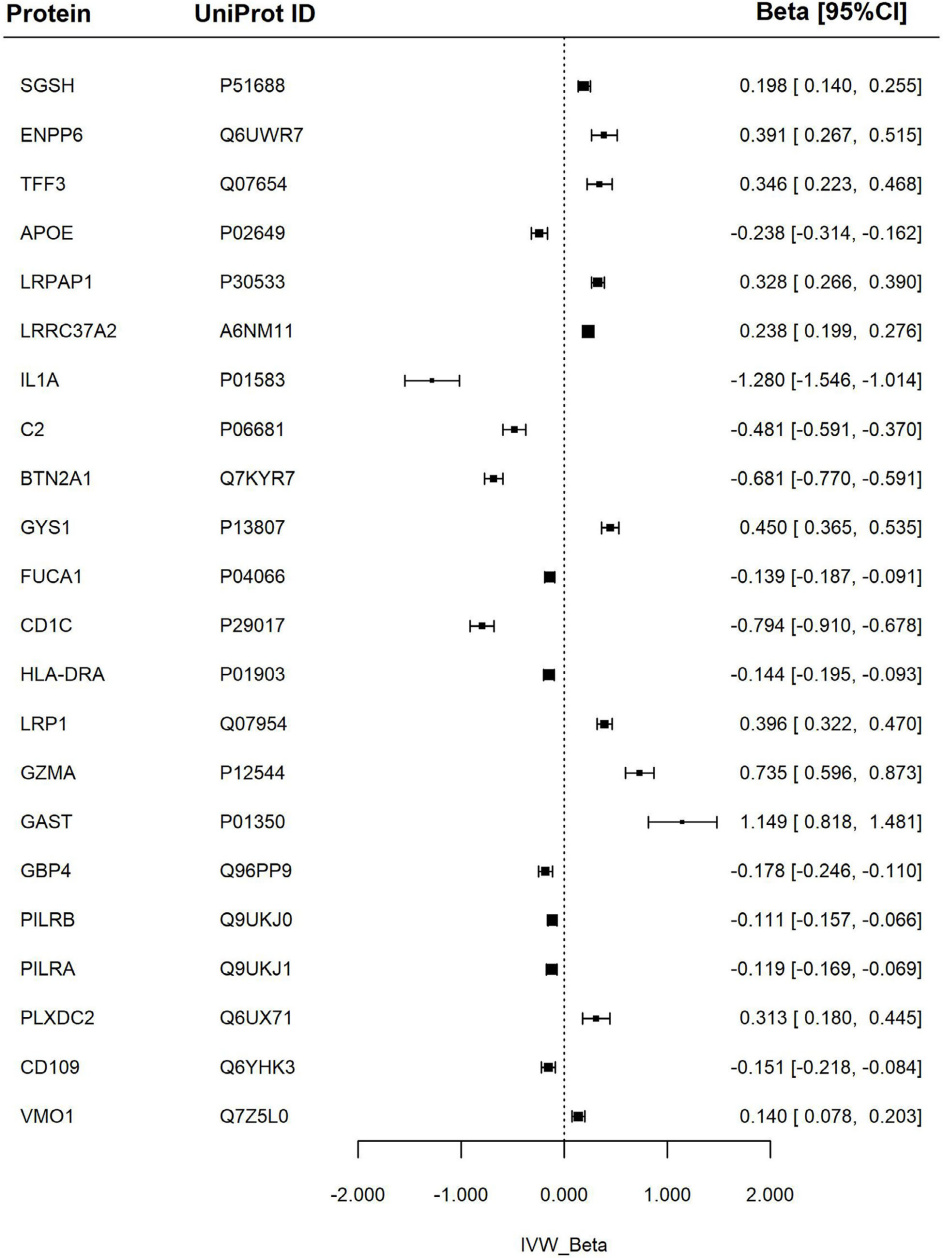
Association between proteins and mean diffusivity. Forest plot of Mendelian randomization estimates for mean diffusivity in relation to specific proteins. The IVW *β* represents the inverse variance weighted effect size with corresponding 95% confidence intervals (*β* [95% CI]). APOE, apolipoprotein E; BTN2A1, butyrophilin subfamily 2 member A1; C2, complement C2; CD109, CD109 antigen; CD1C, T‐cell surface glycoprotein CD1c; ENPP6, glycerophosphocholine choline phosphodiesterase ENPP6; FUCA1, Tissue alpha‐L‐fucosidase; GAST, gastrin; GBP4, guanylate‐binding protein 4; GYS1, glycogen [starch] synthase, muscle; GZMA, granzyme A; HLA‐DRA, HLA class II histocompatibility antigen‐DR alpha chain; IL1A, interleukin‐1 alpha; LRP1, pro‐low‐density lipoprotein receptor‐related protein 1; LRPAP1, alpha‐2‐macroglobulin receptor‐associated protein; LRRC37A2, leucine‐rich repeat‐containing protein 37A2; PILRA, paired immunoglobulin‐like type 2 receptor alpha; PILRB, paired immunoglobulin‐like type 2 receptor beta; PLXDC2, plexin domain‐containing protein 2; SGSH, N‐sulphoglucosamine sulphohydrolase; TFF3, trefoil factor 3; VMO1, vitelline membrane outer layer protein 1 homolog.

Across all neuroimaging biomarkers, > 280 significant associations were identified in the main analysis. However, the number of significant associations decreased after Bonferroni correction for multiple testing. WMHs retained the highest number of significant associations after Bonferroni correction, with 78 associations. After complementary analyses, the number of significant associations was further refined to 35 for WMHs, 22 for MD, and 25 for FA (Figures 3, 4, 5, Tables S3 and S5 in supporting information).

### 3.6 WMHs

The largest effect sizes were associations of higher serum levels of malignant T‐cell‐amplified sequence (MCTS1), procollagen C‐endopeptidase enhancer 1 (PCOLCE), granzyme A (GZMA), and TP53‐binding protein 1 (TP53BP1) with greater (unhealthy) WMHs.

The most prominent negative associations were with protein disulfide‐isomerase A5 (PDIA5), transmembrane protein 25 (TMEM25), T‐cell surface glycoprotein CD1c (CD1C), and lithostathine‐1‐beta (REG1B) levels, which associated with significantly lower WMHs. There was a significant but smaller negative association between apoE and WMHs.

Additional significant results are presented in Figure 3 and Table S5.

### 3.7 FA

The greatest positive associations were observed with serum interleukin‐1 alpha (IL1A), 5‐oxoprolinase (OPLAH), CD1C, and butyrophilin subfamily 2 member A1 (BTN2A1), higher levels of which were linked to higher (healthier) global FA values.

Guanylate‐binding protein 2 (GBP2) showed the greatest negative association with FA. Smaller significant negative associations were observed with GZMA, BTN3A2, and brevican core protein (BCAN) levels.

The full results are presented in Figure 4 and Table S5.

### 3.8 MD

Higher IL1A, CD1C, BTN2A1, and complement component 2 (C2) levels were linked to lower (healthier) global MD values. Higher apoE levels showed similar but smaller magnitude associations with lower MD. The largest magnitude positive associations were with GZMA, gastrin (GAST), glycogen synthase, muscle (GYS1), glycerophosphocholine cholinephosphodiesterase (ENPP6), and trefoil factor 3 (TFF3).

The full results are detailed in Figure 5 and Table S5.

## 4 DISCUSSION

This study provides a high‐throughput screening analysis of the genetic association between plasma proteins and major dementia subtypes and neuroimaging markers that are of likely causal significance. It confirms known associations and uncovers novel proteins potentially involved in disease mechanisms. Notably, it highlights various pathways in lipid metabolism, Aβ processing, immune regulation, inflammation, and cellular growth as key contributors to dementia pathogenesis. In the following sections, we explore the potential functional role of selected proteins associated with dementia subtypes and MRI‐derived phenotypes.

### 4.1 AD

Our analysis showed a consistent association of higher genetically predicted serum apoE levels with lower risk of AD, better white matter MD (lower global MD), and fewer WMHs (lower WMHs). The protective role of apoE is attributed to its high avidity for, and specific binding and clearance of, Aβ peptides.^20^ The links between the *APOE* ɛ4 allele and higher risk of late‐onset familial AD is well established. However, its precise mechanistic role remains debated, whether cognitive deficits in *APOE* ɛ4 carriers are a prodrome of dementia pathology or if they reflect a direct contribution of the *APOE* genotype.^21^ Our analysis suggests a direct mechanistic role between higher apoE levels in the serum and reduced risk of AD and healthier neuroimaging biomarkers. Notably, we also found that higher apoE levels were linked to lower risk of both VD and PDD, although with smaller magnitude of effect, suggesting greater mechanistic specificity with AD.

Higher TREM2 levels were linked to lower risk of AD. TREM2 is a microglial immunoreceptor with loss‐of‐function mutations linked to early‐onset dementia in humans and genetic variants associated with increased risk of neurodegenerative disorders.^22^ In mouse models, TREM2 deficiency has been associated with impaired Aβ breakdown and AD‐related pathological alterations.^19^ Our findings support these previous biological studies and indicate a likely causal link between serum TREM2 levels and AD risk.

Higher serum CD74 levels were associated with a lower risk of AD. CD74 is involved in MHC class II processing and acts as a receptor for macrophage migration inhibitory factor (MIF).^23^ Immunohistochemical data show increased CD74 in AD neurons, particularly in tangles and plaque.^24^ Our results highlight the importance of CD74 in AD pathophysiology.

Higher serum FCRL3 levels were linked to a lower risk of AD in our analysis. FCRL3 is a member of the immunoglobulin receptor superfamily and has a suggested role in immune regulation, although its precise function remains unknown. Its role in autoimmune conditions has been reported.^25^ Our findings suggest protective association of FCRL3 with AD, an association which has not been previously reported.

We additionally found association of higher BTN3A2 levels with higher risk of AD and VD, and with greater WMHs (higher WMHs) and unhealthy brain connectivity (lower FA). The BTN3A2 protein is part of the immunoglobulin superfamily and may be involved in the adaptive immune response.^26^ The *BTN3A2* gene has previously been linked to schizophrenia risk^27^ and greater risk of lacunar strokes.^28^ Our analysis is the first to report associations with increased AD risk.

### 4.2 VD

Higher apoE levels were also protective against VD, although to a lesser extent than with AD. Associations included healthier neuroimaging biomarkers, notably fewer WMHs. Our findings support growing evidence indicating a protective role of apoE in relation to VD,^29^ through maintenance of neurovascular regulation and white matter integrity.^30^

We also identified association of higher serum BTN3A2 levels with increased risk of VD and adverse neuroimaging phenotypes (and higher AD risk, as previously mentioned), corroborating existing evidence in this area.^28^

Higher CNTN2 levels were linked to increased VD risk. The contactin group of proteins are part of the immunoglobulin superfamily of cell adhesion molecules and have a crucial function for maintaining the integrity and function of myelinated axons.^31^ They have also been suggested to interact with amyloid precursor proteins and linked to AD development.^32^ Previous work has demonstrated significant reduction in levels of CNTN2 in cerebrospinal fluid of patients with AD. Our study reports new associations between higher serum CNTN2 and greater risk of VD.

We found association of higher SMAD3 levels with lower risk of VD. SMAD3 is an intracellular signal transducer and transcriptional modulator activated by TGFβ (transforming growth factor beta) and activin type 1 receptor kinases. Biologic studies indicate impairment of the TGFβ–SMAD3 pathway with aging, facilitating cytotoxic activation of microglia and microglia‐mediated neurodegeneration.^33^ While some studies have suggested links between SMAD3 and AD,^34^ ours is the first to report links to VD.

### 4.3 PDD

Higher apoE levels were linked to lower risk of PDD, although the greatest effect size was in relation to AD as previously discussed.

Our results show association of higher serum level of SNAP‐25, a presynaptic protein involved in regulation of neurotransmitter release and synaptic function,^35^ with higher PDD risk and increased WMHs. Previous work has highlighted the relevance of SNAP‐25 in neurodegeneration in the context of PDD and called for its consideration as a biomarker for early diagnosis and novel drug targets.^36^, ^37^, ^38^ Our findings support these previous suggestions.

We identified links between higher TNR levels and increased risk of PDD. TNR is a neural extracellular matrix protein involved in interactions that can influence neurite growth. Recent reports, using whole exome sequencing, have identified TNR as rare variants relevant in familial Parkinson's disease.^39^ Our findings support these earlier results and present serum TNR as a potential biomarker of PDD.

We additionally found novel association of higher MENT protein levels with higher PDD risk and greater WMHs. The function of this protein is not well understood but is believed to have a role in control of cellular proliferation and tumor suppression.^40^ MENT has not been previously linked to cognitive health or dementia. Our findings highlight this protein as a potential biomarker of interest in PDD, which merits further study.

### 4.4 MRI biomarkers and dementia

Higher apoE levels were linked to healthier neuroimaging phenotypes, consistent with existing understanding and demonstrating protective relationships with dementias.

Higher serum levels of GZMA, a protein with proposed roles as a pro‐inflammatory agent and cell death initiator,^41^ were linked to greater white matter micro‐injury (greater WMHs) and degeneration of MD (lower FA, higher MD). Previous work has identified higher serum levels of GZMA in patients with amyotrophic lateral sclerosis.^42^ Our findings suggest GZMA as a potential biomarker as higher serum GZMA levels are linked with adverse neuroimaging indicators.

Higher serum IL1A levels were linked with healthier neuroimaging phenotypes (higher FA, lower MD). Our findings are consistent with previous evidence highlighting the critical role of the IL1 proteins in neuroinflammation and linked polymorphisms of the *IL1A* gene to increased risk of AD.^43^, ^44^

Higher BTN2A1 levels were linked to reduced evidence of WMHs (lower WMHs) and better microstructural integrity. Mutations in the *BTN2A1* gene have been linked to hypertension and metabolic syndrome, and autoimmune conditions.^45^ We demonstrate these relationships with brain health for the first time.

ENPP6 was associated with unhealthy FA and MD changes, indicating deleterious disruption of microstructural white matter integrity. ENPP6 is a choline‐specific phosphodiesterase with a role in choline metabolism,^46^ and has been identified as a risk locus for psychosis in AD.^47^ Our results extend existing knowledge by demonstrating associations of ENPP6 with deleterious alterations of neuroimaging biomarkers.

Higher TFF3 was also linked with adverse imaging markers (higher FA, lower MD). TFF3 is a protein involved in the maintenance of intestinal mucosal integrity with emerging evidence highlighting roles in oncogenesis and regulation of brain function.^48^ Previous work suggests a potential role of TFF3 in predicting PDD or VD in specific contexts.^49^ Our study is the first to suggest a role for TFF3 as a biomarker for detection of early neurodegeneration.

### 4.5 Limitations

The reliance on GWAS datasets predominantly from European populations limits generalizability to other population groups.

While plasma protein levels serve as convenient proxies for systemic biological activity, they may not fully reflect the protein activity within specific tissues, such as the brain. Given that many neurodegenerative processes are localized within the central nervous system, the extrapolation of plasma protein findings to brain‐specific mechanisms must be made with caution. Complementary studies using CSF or brain tissue samples could help validate and refine our conclusions. MR relies on the assumption that the genetic instruments are strongly associated with the exposure, not associated with confounders, and affect the outcome only through the exposure. Additionally, pleiotropy remains a key challenge in MR studies.

Finally, the accuracy of dementia subtyping depends on the methods used to identify cases, with potential risk of misclassification or heterogeneity in diagnostic criteria, which could influence the specificity of our findings. A significant challenge is that diagnoses might not represent a “pure” AD or VD case. Instead, mixed dementia is common. As a result, GWASs based on clinical diagnoses likely reflect a mixed pathological picture rather than distinct disease entities, complicating the interpretation of genetic associations. A recent study highlighted the importance of pathology‐confirmed GWASs or those focused on pathology‐derived endophenotypes to address this issue.^3^ Such approaches provide a more refined understanding of genetic centrifugations and should be considered in future studies.

## CONFLICT OF INTEREST STATEMENT

The authors have nothing to disclose.

## CONSENT STATEMENT

This research used de‐identified, publicly available data from the UK Biobank and FinnGen studies, where all participants had provided informed consent at enrolment. No new recruitment or direct interaction with human subjects was undertaken, and additional consent was therefore not required.

## Supporting information



Supporting Information

Supporting Information

Supporting Information

## Data Availability

The data used to replicate the findings of this study are publicly available. GWAS summary statistics of proteins can be downloaded from [12],^12^ brain diseases from FinnGen [13,14],^13,14^ and brain MRI biomarkers from [15].^15^ All HTML files displaying the results of the current study will be available online at http://mrstudies.org/ [92].^92^

## References

[1] Nichols E , Steinmetz JD , Vollset SE , et al. Estimation of the global prevalence of dementia in 2019 and forecasted prevalence in 2050: an analysis for the Global Burden of Disease Study 2019. The Lancet Public Health. 2022;7(2):e105–e125. 34998485 10.1016/S2468-2667(21)00249-8 PMC8810394

[2] Aarsland D , Kurz MW . The epidemiology of dementia associated with Parkinson disease. J Neurol Sci. 2010;289:18–22. 19733364 10.1016/j.jns.2009.08.034

[3] Scheltens P , De Strooper B , Kivipelto M , et al. Alzheimer's disease. Lancet. 2021;397:1577–1590. 33667416 10.1016/S0140-6736(20)32205-4 PMC8354300

[4] Hampel H , Toschi N , Babiloni C , et al. Revolution of Alzheimer precision neurology. Passageway of systems biology and neurophysiology. J Alzheimer's Dis. 2018;64:S47–S105. 29562524 10.3233/JAD-179932 PMC6008221

[5] De Strooper B , Karran E . The cellular phase of Alzheimer's disease. Cell. 2016;164:603–615. 26871627 10.1016/j.cell.2015.12.056

[6] Bir SC , Khan MW , Javalkar V , Toledo EG , Kelley RE . Emerging concepts in vascular dementia: a review. J Stroke Cerebrovasc Dis. 2021;30:105864. 34062312 10.1016/j.jstrokecerebrovasdis.2021.105864

[7] Zhu Z , Zheng Z , Zhang F , et al. Causal associations between risk factors and common diseases inferred from GWAS summary data. Nat Commun. 2018;9:224. 29335400 10.1038/s41467-017-02317-2 PMC5768719

[8] Davey Smith G , Ebrahim S . Mendelian randomization: can genetic epidemiology contribute to understanding environmental determinants of disease? Int J Epidemiol. 2003;32:1–22. 12689998 10.1093/ije/dyg070

[9] Jansen IE , Savage JE , Watanabe K , et al. Genome‐wide meta‐analysis identifies new loci and functional pathways influencing Alzheimer's disease risk. Nat Genet. 2019;51:404–413. 30617256 10.1038/s41588-018-0311-9 PMC6836675

[10] Sun BB , Suhre K , Gibson BW . Promises and challenges of populational proteomics in health and disease. Mol Cell Proteomics. 2024;23:100786. 38761890 10.1016/j.mcpro.2024.100786 PMC11193116

[11] Skrivankova VW , Richmond RC , Woolf BAR , et al. Strengthening the reporting of observational studies in epidemiology using Mendelian randomization (STROBE‐MR): explanation and elaboration. BMJ. 2021;375:n2233. 34702754 10.1136/bmj.n2233 PMC8546498

[12] Sun BB , Chiou J , Traylor M , et al. Plasma proteomic associations with genetics and health in the UK Biobank. Nature. 2023;622:329–338. 37794186 10.1038/s41586-023-06592-6 PMC10567551

[13] Molecular Medicine Finland (FIMM) . FinnGen: An expedition into genomics and medicine [Internet]. The University of Helsink. [cited 2025 Oct 12]. Available from: https://www.finngen.fi/en

[14] Kurki MI , Karjalainen J , Palta P , et al. FinnGen provides genetic insights from a well‐phenotyped isolated population. Nature. 2023;613:508–518. 36653562 10.1038/s41586-022-05473-8 PMC9849126

[15] Persyn E , Hanscombe KB , Howson JMM , et al. Genome‐wide association study of MRI markers of cerebral small vessel disease in 42,310 participants. Nat Commun. 2020;11:2175. 32358547 10.1038/s41467-020-15932-3 PMC7195435

[16] Prins ND , Scheltens P . White matter hyperintensities, cognitive impairment and dementia: an update. Nat Rev Neurol. 2015;11(3):157–165. 25686760 10.1038/nrneurol.2015.10

[17] Power MC , Su D , Wu A , et al. Association of white matter microstructural integrity with cognition and dementia. Neurobiol Aging. 2019;83:63. 31585368 10.1016/j.neurobiolaging.2019.08.021 PMC6914220

[18] Bowden J , Davey Smith G , Burgess S . Mendelian randomization with invalid instruments: effect estimation and bias detection through Egger regression. Int J Epidemiol. 2015;44:512–525. 26050253 10.1093/ije/dyv080 PMC4469799

[19] Zhao Y , Wu X , Li X , et al. TREM2 is a receptor for β‐amyloid that mediates microglial function. Neuron. 2018;97:1023–1031.e7. 29518356 10.1016/j.neuron.2018.01.031 PMC5889092

[20] Huang YWA , Zhou B , Wernig M , Südhof TC . ApoE2, ApoE3, and ApoE4 differentially stimulate APP transcription and Aβ secretion. Cell. 2017;168:427–441.e21. 28111074 10.1016/j.cell.2016.12.044 PMC5310835

[21] O'Donoghue MC , Murphy SE , Zamboni G , Nobre AC , Mackay CE . APOE genotype and cognition in healthy individuals at risk of Alzheimer's disease: a review. Cortex. 2018;104:103–123. 29800787 10.1016/j.cortex.2018.03.025

[22] Cantoni C , Bollman B , Licastro D , et al. TREM2 regulates microglial cell activation in response to demyelination in vivo. Acta Neuropathol. 2015;129:429. 25631124 10.1007/s00401-015-1388-1 PMC4667728

[23] Becker‐Herman S , Arie G , Medvedovsky H , Kerem A , Shachar I . CD74 is a member of the regulated intramembrane proteolysis‐processed protein family. Mol Biol Cell. 2005;16:5061. 16107560 10.1091/mbc.E05-04-0327 PMC1266406

[24] Bryan KJ , Zhu X , Harris PL , et al. Expression of CD74 is increased in neurofibrillary tangles in Alzheimer's disease. Mol Neurodegener. 2008;3:13. 18786268 10.1186/1750-1326-3-13 PMC2565661

[25] Matesanz F , Fernández O , Milne RL , et al. The high producer variant of the Fc‐receptor like‐3 (FCRL3) gene is involved in protection against multiple sclerosis. J Neuroimmunol. 2008;195:146–150. 18313765 10.1016/j.jneuroim.2008.01.004

[26] BTN3A2 butyrophilin subfamily 3 member A2 [Homo sapiens (human)]—Gene—NCBI. https://www.ncbi.nlm.nih.gov/gene?Db=gene&Cmd=DetailsSearch&Term=11118

[27] Wu Y , Bi R , Zeng C , et al. Identification of the primate‐specific gene BTN3A2 as an additional schizophrenia risk gene in the MHC loci. EBioMedicine. 2019;44:530. 31133542 10.1016/j.ebiom.2019.05.006 PMC6603853

[28] Yang X‐Z , Huang M‐Y , Han F , et al. Genome‐wide Mendelian randomization study reveals druggable genes for cerebral small vessel disease. Stroke. 2024;55:2264–2273. 39114924 10.1161/STROKEAHA.124.046544

[29] Rohn TT . Is apolipoprotein E4 an important risk factor for vascular dementia? Int J Clin Exp Pathol. 2014;7:3504. 25120729 PMC4128964

[30] Koizumi K , Hattori Y , Ahn SJi , et al. Apoε4 disrupts neurovascular regulation and undermines white matter integrity and cognitive function. Nat Commun. 2018;9(1):1–11. 30232327 10.1038/s41467-018-06301-2 PMC6145902

[31] Kalafatakis I , Savvaki M , Velona T , Karagogeos D . Implication of contactins in demyelinating pathologies. Life. 2021;11:51. 33451101 10.3390/life11010051 PMC7828632

[32] Bamford RA , Widagdo J , Takamura N , et al. The interaction between contactin and amyloid precursor protein and its role in Alzheimer's disease. Neuroscience. 2020;424:184–202. 31705890 10.1016/j.neuroscience.2019.10.006

[33] Tichauer JE , Flores B , Soler B , et al. Age‐dependent changes on TGFβ1 SMAD3 pathway modify the pattern of microglial cell activation. Brain Behav Immun. 2013;37:187. 24380849 10.1016/j.bbi.2013.12.018 PMC3951654

[34] Chalmers KA , Love S . Neurofibrillary tangles may interfere with SMAD 2/3 signaling in neurons. J Neuropathol Exp Neurol. 2007;66:158–167. 17279001 10.1097/nen.0b013e3180303b93

[35] Wolner SH , Gleerup HS , Musaeus CS , et al. Synaptosomal‐associated protein 25 kDA (SNAP‐25) levels in cerebrospinal fluid: implications for Alzheimer's disease diagnosis and monitoring. Synapse. 2025;79:e70010. 39912369 10.1002/syn.70010 PMC11800177

[36] Bereczki E , Bogstedt A , Höglund K , et al. Synaptic proteins in CSF relate to Parkinson's disease stage markers. npj Parkinson's Dis. 2017;3(1):1–5. 28649607 10.1038/s41531-017-0008-2 PMC5445607

[37] Zhang C , Xie S , Malek M . SNAP‐25: a biomarker of synaptic loss in neurodegeneration. Clin Chim Acta. 2025;571:120236. 40058720 10.1016/j.cca.2025.120236

[38] Wang Q , Tao S , Xing L , et al. SNAP‐25 is a potential target for early stage Alzheimer's disease and Parkinson's disease. Eur J Med Res. 2023;28:570. 38053192 10.1186/s40001-023-01360-8 PMC10699008

[39] Farlow JL , Robak LA , Hetrick K , et al. Whole‐exome sequencing in familial Parkinson disease. JAMA Neurol. 2016;73:68‐75. 26595808 10.1001/jamaneurol.2015.3266 PMC4946647

[40] Hlady RA , Novakova S , Opavska J , et al. Loss of Dnmt3b function upregulates the tumor modifier Ment and accelerates mouse lymphomagenesis. J Clin Invest. 2012;122:163–177. 22133874 10.1172/JCI57292 PMC3248285

[41] Zhou Z , He H , Wang K , et al. Granzyme A from cytotoxic lymphocytes cleaves GSDMB to trigger pyroptosis in target cells. Science. 2020;368:eaaz7548. 32299851 10.1126/science.aaz7548

[42] Iłz̈ecka J . Granzymes A and B levels in serum of patients with amyotrophic lateral sclerosis. Clin Biochem. 2011;44:650–653. 21349256 10.1016/j.clinbiochem.2011.02.006

[43] Mrak R . Interleukin‐1, neuroinflammation, and Alzheimer's disease. Neurobiol Aging. 2001;22:903–908. 11754997 10.1016/s0197-4580(01)00287-1

[44] Shaftel SS , Griffin WST , O'Banion MK . The role of Interleukin‐1 in neuroinflammation and Alzheimer disease: an evolving perspective. J Neuroinflammation. 2008;5:7. 18302763 10.1186/1742-2094-5-7 PMC2335091

[45] Oguri M , Kato K , Yoshida T , et al. Association of a genetic variant of BTN2A1 with metabolic syndrome in East Asian populations. J Med Genet 2011;48(11):787–792. 21784758 10.1136/jmg.2010.088138

[46] Morita J , Kano K , Kato K , et al. Structure and biological function of ENPP6, a choline‐specific glycerophosphodiester‐phosphodiesterase. Sci Rep. 2016;6(1):1–14. 26888014 10.1038/srep20995 PMC4757880

[47] DeMichele‐Sweet MAA , Klei L , Creese B , et al. Genome‐wide association identifies the first risk loci for psychosis in Alzheimer disease. Mol Psychiatry. 2021;26:5797. 34112972 10.1038/s41380-021-01152-8 PMC8660923

[48] Yang Y , Lin Z , Lin Q , Bei W , Guo J . Pathological and therapeutic roles of bioactive peptide Trefoil factor 3 in diverse diseases: recent progress and perspective. Cell Death Dis. 2022;13(1):1–14. 10.1038/s41419-022-04504-6 PMC8763889 35039476

[49] Zou J , Chen Z , Liang C , et al. Trefoil factor 3, cholinesterase and homocysteine: potential predictors for Parkinson's disease dementia and vascular Parkinsonism dementia in advanced stage. Aging Dis. 2018;9:51. 29392081 10.14336/AD.2017.0416 PMC5772858

