# Supplementary material for: Genetic associations of plasma proteomics with dementia subtypes and neuroimaging markers
Source: Alzheimers Dement (Amst). 2025 Oct 21;17(4):e70202. doi: 10.1002/dad2.70202 (PMC12538646; doi:10.1002/dad2.70202)
Supplement: Supplementary file 1 — Supporting Information [file DAD2-17-e70202-s003.docx]

**Supplementary Material**

**Genetic associations of plasma proteomic with dementia subtypes and neuroimaging markers**

[Supplementary Table 1: Summary of genome-wide association studies for dementia subtypes 2](#_Toc204701660)

[Supplementary Table 2: Summary of genome-wide association studies for neuroimaging biomarkers 3](#_Toc204701661)

[Supplementary Table 3: Number of significant associations between proteins and outcomes in the main analysis, adjusted for multiple tests and in the complementary analyses 4](#_Toc204701662)

[Supplementary Table 4: List of the significant associations between proteins and dementia subtypes 5](#_Toc204701663)

[Supplementary Table 5: List of the significant associations between proteins and MRI biomarkers 7](#_Toc204701664)

[Supplementary Table 6: Summary of Case and Control Definitions for Registry-Based Dementia Diagnoses 12](#_Toc204701665)

[Supplementary Figure 1: Scatter plot showing the pattern of significance in the relationship between proteins and various diseases as well as MRI biomarkers 13](#_Toc204701666)

[References 16](#_Toc204701667)

# Supplementary Table 1: Summary of genome-wide association studies for dementia subtypes

| **Outcome** | **Reference** | **Cohort** | **Cases** | **Control** | **Sample size** | **GWAS summary statistics link** |
| --- | --- | --- | --- | --- | --- | --- |
| Vascular Dementia | Kurki MI et al. (1) | FinnGen | 2,667 | 426,542 | 429,209 | <https://r10.risteys.finregistry.fi/endpoints/F5_VASCDEM> |
| Alzheimer Disease |  |  | 12,348 | 416,861 | 429,209 | <https://r10.risteys.finngen.fi/endpoints/G6_ALZHEIMER_INCLAVO> |
| Parkinson`s Disaese |  |  | 589 | 428,620 | 429,209 | <https://r10.risteys.finngen.fi/endpoints/PD_DEMENTIA#summary-statistics> |

**Footnote Supplementary Table 1:**

The table summarises the genome-wide association studies used for brain diseases in the MR analysis. GWAS - genome wide association studies.

# Supplementary Table 2: Summary of Case and Control Definitions for Registry-Based Dementia Diagnoses

| **Diseases** | **Cases** | **Control** |
| --- | --- | --- |
| Vascular Dementia | Hospital discharge [ICD-10 (F01), ICD-9 (4378)]  Cause of death [ICD-10 (F01), ICD-9 (4378)] | Free of:  Hospital discharge [ICD-10 (F00-F09), ICD-9 (290\|3310\|4378A), ICD-8 (290)]  Cause of death [ICD-10 (F00-F09), ICD-9 (290\|3310\|4378A), ICD-8 (290)]  KELA reimbursements [KELA codes (307)]  Medicine purchases [ATC (N06D)] |
| Alzheimer disease | Primary healthcare outpatient [ICD-10 (G30)] | Individuals that are not cases. |
| Dementia due to Parkinson's Disease | Hospital discharge [ICD-10 (F02.3, F02.39)]  Cause of death [ICD-10 (F02.3, F02.39)] | Free of:  Hospital discharge [ICD-10 (G20), ICD-9 (3320A), ICD-8 (34200)]  Cause of death [ICD-10 (G20), ICD-9 (3320A), ICD-8 (34200)]  KELA reimbursements [KELA codes (110)] |

**Footnote for supplementary table 2**:

Diagnoses were ascertained using national healthcare registers, including hospital discharge records, causes of death, KELA reimbursement data, medicine purchases, and primary healthcare outpatient records. International Classification of Diseases (ICD) codes, ATC codes, and KELA reimbursement codes were used to classify cases. Controls were defined as individuals without any corresponding diagnostic or treatment codes indicative of the respective dementia subtype

# Supplementary Table 3: Summary of genome-wide association studies for neuroimaging biomarkers

| **MRI metrics** | **Reference** | **Cohort** | **Sample size** | **GWAS summary statistics link** |
| --- | --- | --- | --- | --- |
| WMH | Persyn E et al. (2) | UK Biobank | 42,310 | <https://kp4cd.org/node/1071> |
| FA |  |  | 17,663 |  |
| MD |  |  | 17,467 |  |

**Footnote Supplementary Table 3:**

The table summarises the genome-wide association studies used for neuroimaging biomarkers in the MR analysis. FA - Fractional Anisotropy; MD - Mean Diffusivity; WMH - White Matter Hyperintensities.

# Supplementary Table 4: Number of significant associations between proteins and outcomes in the main analysis, adjusted for multiple tests and in the complementary analyses

| **Outcome** | **IVW p-value** | **Corrected p-value (Bonf)** | **Significant in all analyses** |
| --- | --- | --- | --- |
| AD | 360 | 23 | 10 |
| VD | 381 | 21 | 6 |
| PD | 309 | 12 | 11 |
| WMH | 455 | 77 | 35 |
| MD | 540 | 98 | 22 |
| FA | 544 | 80 | 25 |

**Footnote Supplementary Table 4:**

List the significant associations for each outcome that passed the main analysis, the complementary analyses and after adjusting the p-values (IVW) for multiple tests. AD - Alzheimer’s Disease; FA - Fractional Anisotropy; Bonf - Bonferroni; IVW - Inverse-Variance Weighted; MD - Mean Diffusivity; PD - Parkinson’s Disease; VD - Vascular Dementia; WMH - White Matter Hyperintensities.

# Supplementary Table 5: List of the significant associations between proteins and dementia subtypes

| **Protein** | **UniProt ID** | **nsnp** | **MR_Egger**  **pval** | **Wmedia**  **pval** | **IVW**  **beta** | **IVW**  **SE** | **IVW**  **pval** | **Wmode**  **pval** | **Egger_inter**  **pval** | **p-value**  **Bonf** | **MR_P**  **(global)** | **MR_P**  **(distor)** |
| --- | --- | --- | --- | --- | --- | --- | --- | --- | --- | --- | --- | --- |
| **Alzheimer’s disease** | | | | | | | | | | | | |
| CD74 | P04233 | 89 | 8.12E-04 | 2.00E-07 | -2.25E-01 | 3.22E-02 | 0.00E+00 | 1.83E-03 | 7.34E-01 | 0.00E+00 | <0.0025 |  |
| APOE | P02649 | 92 | 0.00E+00 | 0.00E+00 | -6.91E-01 | 5.77E-02 | 0.00E+00 | 0.00E+00 | 6.49E-01 | 0.00E+00 | <0.0025 | 6.40E-01 |
| BTN3A2 | P78410 | 211 | 3.30E-05 | 1.60E-06 | 6.47E-02 | 1.13E-02 | 0.00E+00 | 1.52E-04 | 1.56E-01 | 0.00E+00 | <0.0025 | 9.13E-01 |
| FCRL3 | Q96P31 | 136 | 1.11E-04 | 2.00E-07 | -6.99E-02 | 1.25E-02 | 0.00E+00 | 0.00E+00 | 5.92E-01 | 0.00E+00 | 3.00E-02 |  |
| TREM2 | Q9NZC2 | 83 | 2.64E-04 | 2.50E-06 | -1.87E-01 | 3.02E-02 | 0.00E+00 | 1.54E-05 | 4.67E-01 | 0.00E+00 | <0.0025 | 3.58E-01 |
| PILRA | Q9UKJ1 | 152 | 5.00E-07 | 8.94E-05 | 5.60E-02 | 9.11E-03 | 0.00E+00 | 5.59E-05 | 1.26E-01 | 0.00E+00 | 2.25E-01 |  |
| CD55 | P08174 | 61 | 2.68E-04 | 2.80E-06 | -1.57E-01 | 2.31E-02 | 0.00E+00 | 1.09E-05 | 5.75E-01 | 0.00E+00 | 3.85E-01 |  |
| LRRC25 | Q8N386 | 138 | 4.68E-02 | 2.73E-04 | -9.60E-02 | 2.01E-02 | 1.90E-06 | 1.04E-03 | 5.07E-01 | 4.13E-03 | 4.75E-02 | 7.90E-01 |
| CD86 | P42081 | 56 | 2.75E-03 | 1.80E-04 | -1.44E-01 | 3.11E-02 | 3.50E-06 | 9.14E-03 | 4.55E-01 | 7.60E-03 | 8.53E-01 |  |
| TNXB | P22105 | 152 | 5.67E-04 | 1.83E-04 | -7.63E-02 | 1.69E-02 | 6.20E-06 | 8.41E-04 | 3.36E-01 | 1.35E-02 | 5.00E-03 |  |
| **Vascular dementia** | | | | | | | | | | | | |
| SPARCL1 | Q14515 | 123 | 3.58E-04 | 4.21E-03 | -1.40E-01 | 2.49E-02 | 0.00E+00 | 7.48E-05 | 8.56E-01 | 0.00E+00 | 8.00E-02 |  |
| APOE | P02649 | 92 | 0.00E+00 | 0.00E+00 | -4.05E-01 | 4.27E-02 | 0.00E+00 | 0.00E+00 | 8.98E-01 | 0.00E+00 | <0.0025 | 2.45E-01 |
| BTN3A2 | P78410 | 211 | 1.49E-02 | 8.00E-06 | 1.15E-01 | 1.77E-02 | 0.00E+00 | 2.06E-04 | 2.42E-01 | 0.00E+00 | 7.65E-01 |  |
| FBN2 | P35556 | 18 | 1.76E-03 | 2.09E-05 | -3.93E-01 | 7.88E-02 | 6.00E-07 | 7.09E-04 | 3.51E-01 | 1.30E-03 | 7.35E-01 |  |
| CNTN2 | Q02246 | 104 | 2.04E-04 | 2.56E-02 | 1.03E-01 | 2.24E-02 | 4.40E-06 | 3.53E-03 | 3.28E-01 | 9.56E-03 | 9.28E-01 |  |
| SMAD3 | P84022 | 19 | 1.30E-02 | 3.49E-03 | -3.48E-01 | 7.73E-02 | 6.60E-06 | 5.33E-03 | 9.65E-01 | 1.43E-02 | 9.38E-01 |  |
| **Parkinson’s disease** | | | | | | | | | | | | |
| MENT | Q9BUN1 | 84 | 1.88E-03 | 1.28E-03 | 4.80E-01 | 8.61E-02 | 0.00E+00 | 1.12E-04 | 6.96E-01 | 0.00E+00 | 2.28E-01 |  |
| IL19 | Q9UHD0 | 67 | 3.30E-02 | 1.18E-02 | -5.25E-01 | 9.04E-02 | 0.00E+00 | 2.19E-02 | 4.88E-01 | 0.00E+00 | 5.40E-01 |  |
| APOE | P02649 | 92 | 7.85E-05 | 4.93E-04 | -3.05E-01 | 5.72E-02 | 1.00E-07 | 5.08E-04 | 3.47E-01 | 2.17E-04 | 4.10E-01 |  |
| IL6R | P08887 | 105 | 8.26E-05 | 3.06E-03 | -1.89E-01 | 3.88E-02 | 1.20E-06 | 2.87E-03 | 4.08E-01 | 2.61E-03 | 4.25E-01 |  |
| SNAP25 | P60880 | 56 | 3.96E-05 | 3.00E-06 | 5.18E-01 | 1.08E-01 | 1.80E-06 | 5.20E-06 | 7.31E-02 | 3.91E-03 | 2.50E-03 | 7.38E-01 |
| TNR | Q92752 | 110 | 1.69E-02 | 2.12E-03 | 3.80E-01 | 8.10E-02 | 2.70E-06 | 1.81E-03 | 6.57E-01 | 5.86E-03 | 9.48E-01 |  |
| HPGDS | O60760 | 55 | 5.58E-03 | 1.23E-02 | -3.31E-01 | 7.47E-02 | 9.50E-06 | 2.23E-03 | 5.13E-01 | 2.06E-02 | 6.65E-01 |  |
| MST1 | P26927 | 188 | 8.79E-05 | 5.26E-04 | -1.60E-01 | 3.65E-02 | 1.12E-05 | 3.27E-04 | 2.73E-01 | 2.43E-02 | 5.15E-01 |  |
| VNN2 | O95498 | 103 | 7.98E-04 | 4.22E-02 | -2.34E-01 | 5.35E-02 | 1.24E-05 | 4.87E-03 | 7.24E-01 | 2.69E-02 | 5.33E-01 |  |
| PXN | P49023 | 84 | 1.90E-02 | 2.64E-02 | -2.68E-01 | 6.16E-02 | 1.36E-05 | 8.89E-03 | 5.84E-01 | 2.95E-02 | 6.85E-01 |  |
| SFRP4 | Q6FHJ7 | 37 | 2.04E-02 | 2.27E-03 | 6.32E-01 | 1.48E-01 | 2.05E-05 | 9.80E-03 | 8.03E-01 | 4.45E-02 | 9.88E-01 |  |

**Footnote for supplementary table 5**:

Bonf – corrected IVW p-values; IVW – Inverse variance weighted; MR_P – MR_PRESSO; Nsnp – number of SNPs; SE – standard error; Wmedia – Weighted median; Wmode – Weighted mode; HLA class II histocompatibility antigen gamma chain: CD74; Apolipoprotein E: APOE; Butyrophilin subfamily 3 member A2: BTN3A2; Fc receptor-like protein 3: FCRL3; Triggering receptor expressed on myeloid cells 2: TREM2; Paired immunoglobulin-like type 2 receptor alpha: PILRA; Complement decay-accelerating factor: CD55; Leucine-rich repeat-containing protein 25: LRRC25; T-lymphocyte activation antigen: CD86; Tenascin-X: TNXB; SPARC-like protein 1: SPARCL1; Fibrillin-2: FBN2; Contactin-2: CNTN2; Mothers against decapentaplegic homolog 3:SMAD3; Protein MENT: MENT; Interleukin-19: IL19; Interleukin-6 receptor subunit alpha: IL6R; Synaptosomal-associated protein 25: SNAP25; Tenascin-R: TNR; Hematopoietic prostaglandin D synthase: HPGDS; Hepatocyte growth factor-like protein: MST1; Pantetheine hydrolase VNN2: VNN2; Paxillin: PXN; Secreted frizzled-related protein 4: SFRP4

# Supplementary Table 6: List of the significant associations between proteins and MRI biomarkers

| **Protein** | **UniProt ID** | **nsnp** | **MR_Egger**  **pval** | **Wmedia**  **pval** | **IVW**  **beta** | **IVW**  **SE** | **IVW**  **pval** | **Wmode**  **pval** | **Egger_inter**  **pval** | **p-value**  **Bonf** | **MR_P**  **(global)** | **MR_P**  **(distor)** |
| --- | --- | --- | --- | --- | --- | --- | --- | --- | --- | --- | --- | --- |
| **White matter hyperintensities** | | | | | | | | | | | | |
| TP53BP1 | Q12888 | 66 | 2.07E-03 | 7.00E-06 | 1.25E-01 | 1.87E-02 | 0.00E+00 | 1.98E-02 | 2.38E-01 | 0.00E+00 | 6.68E-01 |  |
| PCOLCE | Q15113 | 7 | 3.81E-02 | 3.70E-06 | 2.10E-01 | 3.65E-02 | 0.00E+00 | 4.89E-03 | 8.74E-01 | 0.00E+00 | 8.83E-01 |  |
| KIR2DL3 | P43628 | 302 | 1.17E-05 | 0.00E+00 | -5.05E-02 | 5.41E-03 | 0.00E+00 | 3.50E-06 | 7.48E-01 | 0.00E+00 | <0.0025 |  |
| BTN2A1 | Q7KYR7 | 208 | 4.51E-04 | 0.00E+00 | -5.88E-02 | 8.11E-03 | 0.00E+00 | 3.89E-05 | 5.93E-01 | 0.00E+00 | 2.50E-01 |  |
| LRRC37A2 | A6NM11 | 218 | 1.20E-06 | 0.00E+00 | 3.75E-02 | 4.52E-03 | 0.00E+00 | 0.00E+00 | 1.68E-01 | 0.00E+00 | <0.0025 | 4.13E-01 |
| BTN3A2 | P78410 | 298 | 3.21E-05 | 0.00E+00 | 4.21E-02 | 4.58E-03 | 0.00E+00 | 3.30E-06 | 4.95E-01 | 0.00E+00 | 5.73E-01 |  |
| CD1C | P29017 | 198 | 1.01E-03 | 5.00E-07 | -7.87E-02 | 1.07E-02 | 0.00E+00 | 2.49E-03 | 9.13E-01 | 0.00E+00 | 1.23E-01 |  |
| GZMA | P12544 | 156 | 1.00E-07 | 0.00E+00 | 1.29E-01 | 1.04E-02 | 0.00E+00 | 1.00E-07 | 4.62E-01 | 0.00E+00 | 5.63E-01 |  |
| SNAP25 | P60880 | 61 | 0.00E+00 | 2.00E-07 | 1.32E-01 | 1.44E-02 | 0.00E+00 | 7.10E-06 | 2.95E-01 | 0.00E+00 | 5.75E-01 |  |
| CPVL | Q9H3G5 | 335 | 3.27E-02 | 3.68E-03 | -2.89E-02 | 5.24E-03 | 0.00E+00 | 2.86E-02 | 1.11E-01 | 0.00E+00 | 2.00E-02 | 7.63E-01 |
| PDIA5 | Q14554 | 148 | 4.14E-02 | 1.70E-06 | -1.04E-01 | 1.19E-02 | 0.00E+00 | 3.78E-03 | 1.12E-01 | 0.00E+00 | 2.25E-02 | 9.13E-01 |
| MCTS1 | Q9ULC4 | 28 | 3.42E-02 | 0.00E+00 | 2.23E-01 | 3.31E-02 | 0.00E+00 | 3.11E-03 | 9.96E-01 | 0.00E+00 | 9.43E-01 |  |
| KIR2DL2 | P43627 | 310 | 2.34E-05 | 2.00E-07 | -4.55E-02 | 5.25E-03 | 0.00E+00 | 7.13E-05 | 8.26E-01 | 0.00E+00 | 7.50E-02 |  |
| MENT | Q9BUN1 | 94 | 9.23E-03 | 6.05E-05 | 7.24E-02 | 1.28E-02 | 0.00E+00 | 5.71E-03 | 3.83E-01 | 0.00E+00 | 3.30E-01 |  |
| HLA-E | P13747 | 233 | 2.87E-02 | 5.10E-06 | -5.08E-02 | 6.42E-03 | 0.00E+00 | 8.58E-04 | 1.31E-01 | 0.00E+00 | 5.50E-02 |  |
| DDR1 | Q08345 | 160 | 2.56E-02 | 3.80E-04 | 8.24E-02 | 1.13E-02 | 0.00E+00 | 7.78E-03 | 7.47E-02 | 0.00E+00 | 5.00E-03 | 7.53E-01 |
| APOE | P02649 | 100 | 7.30E-06 | 9.19E-05 | -5.96E-02 | 9.09E-03 | 0.00E+00 | 4.89E-04 | 4.70E-01 | 0.00E+00 | 2.33E-01 |  |
| TMEM25 | Q86YD3 | 34 | 5.76E-05 | 2.30E-06 | -9.06E-02 | 1.71E-02 | 1.00E-07 | 2.97E-05 | 1.33E-01 | 2.17E-04 | 4.55E-01 |  |
| ASPN | Q9BXN1 | 47 | 7.20E-03 | 7.79E-03 | 1.09E-01 | 2.08E-02 | 2.00E-07 | 1.45E-02 | 6.91E-01 | 4.34E-04 | 1.30E-01 |  |
| TCTN3 | Q6NUS6 | 130 | 2.37E-04 | 3.04E-04 | 4.79E-02 | 9.17E-03 | 2.00E-07 | 6.20E-03 | 7.04E-01 | 4.34E-04 | 6.00E-02 |  |
| PRTG | Q2VWP7 | 117 | 1.84E-02 | 2.00E-02 | 5.83E-02 | 1.13E-02 | 3.00E-07 | 3.06E-03 | 1.80E-01 | 6.51E-04 | 1.48E-01 |  |
| REG1B | P48304 | 85 | 7.65E-03 | 7.53E-04 | -6.21E-02 | 1.24E-02 | 6.00E-07 | 2.44E-03 | 9.14E-01 | 1.30E-03 | 4.33E-01 |  |
| SCARF2 | Q96GP6 | 42 | 8.67E-05 | 1.39E-04 | 8.93E-02 | 1.83E-02 | 1.00E-06 | 2.04E-04 | 8.42E-02 | 2.17E-03 | 9.15E-01 |  |
| LRPAP1 | P30533 | 235 | 1.84E-03 | 3.28E-04 | 2.66E-02 | 5.66E-03 | 2.50E-06 | 3.06E-03 | 7.85E-01 | 5.42E-03 | 4.25E-02 |  |
| COMT | P21964 | 58 | 4.47E-03 | 7.97E-04 | -5.86E-02 | 1.28E-02 | 5.10E-06 | 3.74E-04 | 6.02E-01 | 1.11E-02 | 9.28E-01 |  |
| DKKL1 | Q9UK85 | 165 | 5.33E-03 | 4.84E-03 | 2.44E-02 | 5.37E-03 | 5.60E-06 | 9.04E-04 | 1.79E-01 | 1.21E-02 | 7.00E-01 |  |
| LRRN1 | Q6UXK5 | 116 | 1.22E-03 | 1.84E-02 | -3.63E-02 | 8.06E-03 | 6.90E-06 | 9.48E-04 | 7.16E-01 | 1.50E-02 | 8.28E-01 |  |
| LILRB1 | Q8NHL6 | 253 | 4.63E-02 | 3.37E-02 | 3.63E-02 | 8.08E-03 | 7.00E-06 | 4.23E-02 | 3.58E-01 | 1.52E-02 | <0.0025 | 8.73E-01 |
| HYAL1 | Q12794 | 36 | 1.16E-02 | 2.51E-04 | 1.07E-01 | 2.38E-02 | 7.30E-06 | 5.62E-04 | 6.89E-01 | 1.58E-02 | 1.23E-01 |  |
| LTBP3 | Q9NS15 | 74 | 2.73E-04 | 1.46E-04 | 5.57E-02 | 1.26E-02 | 9.80E-06 | 1.00E-03 | 2.26E-01 | 2.13E-02 | 9.48E-01 |  |
| MTHFSD | Q2M296 | 59 | 7.52E-03 | 4.92E-02 | -5.16E-02 | 1.19E-02 | 1.38E-05 | 1.40E-02 | 8.90E-01 | 2.99E-02 | 5.20E-01 |  |
| PTGR1 | Q14914 | 79 | 2.32E-04 | 9.14E-03 | 4.42E-02 | 1.02E-02 | 1.57E-05 | 7.43E-03 | 1.77E-01 | 3.41E-02 | 9.98E-01 |  |
| TNFSF12 | O43508 | 91 | 7.66E-03 | 2.10E-02 | 5.77E-02 | 1.35E-02 | 1.82E-05 | 2.89E-02 | 7.25E-01 | 3.95E-02 | 4.05E-01 |  |
| RGMB | Q6NW40 | 41 | 1.16E-02 | 2.58E-03 | 1.02E-01 | 2.39E-02 | 1.83E-05 | 1.58E-03 | 4.10E-01 | 3.97E-02 | 4.95E-01 |  |
| HRG | P04196 | 60 | 1.07E-02 | 7.77E-04 | 5.25E-02 | 1.24E-02 | 2.20E-05 | 1.09E-03 | 9.69E-01 | 4.77E-02 | 6.45E-01 |  |
| **Fractional anisotropy** | | | | | | | | | | | | |
| HLA-E | P13747 | 233 | 1.21E-05 | 0.00E+00 | 3.45E-01 | 3.04E-02 | 0.00E+00 | 3.50E-06 | 5.02E-01 | 0.00E+00 | <0.0025 | 9.68E-01 |
| BCAN | Q96GW7 | 58 | 3.61E-03 | 0.00E+00 | -6.84E-01 | 8.69E-02 | 0.00E+00 | 4.30E-06 | 3.84E-01 | 0.00E+00 | 5.38E-01 |  |
| KIR2DL3 | P43628 | 302 | 6.27E-04 | 0.00E+00 | 2.31E-01 | 2.62E-02 | 0.00E+00 | 0.00E+00 | 2.35E-01 | 0.00E+00 | <0.0025 | 6.75E-01 |
| BTN3A2 | P78410 | 298 | 0.00E+00 | 0.00E+00 | -3.46E-01 | 2.09E-02 | 0.00E+00 | 0.00E+00 | 2.68E-01 | 0.00E+00 | 1.13E-01 |  |
| GZMA | P12544 | 156 | 9.90E-06 | 0.00E+00 | -5.86E-01 | 5.67E-02 | 0.00E+00 | 0.00E+00 | 6.85E-01 | 0.00E+00 | <0.0025 | 9.53E-01 |
| LRP1 | Q07954 | 219 | 0.00E+00 | 0.00E+00 | -3.13E-01 | 3.14E-02 | 0.00E+00 | 0.00E+00 | 2.34E-01 | 0.00E+00 | <0.0025 | 7.65E-01 |
| IL1A | P01583 | 24 | 1.95E-04 | 0.00E+00 | 9.66E-01 | 1.33E-01 | 0.00E+00 | 9.95E-05 | 3.73E-01 | 0.00E+00 | 8.40E-01 |  |
| GBP2 | P32456 | 5 | 3.22E-02 | 8.60E-06 | -1.63E+00 | 2.95E-01 | 0.00E+00 | 1.66E-02 | 2.35E-01 | 0.00E+00 | 5.90E-01 |  |
| BTN2A1 | Q7KYR7 | 208 | 2.00E-07 | 0.00E+00 | 5.01E-01 | 4.21E-02 | 0.00E+00 | 0.00E+00 | 6.94E-01 | 0.00E+00 | <0.0025 | 8.38E-01 |
| UROD | P06132 | 82 | 1.42E-04 | 2.00E-07 | -3.36E-01 | 5.10E-02 | 0.00E+00 | 8.60E-06 | 5.61E-01 | 0.00E+00 | 6.70E-01 |  |
| ENPP6 | Q6UWR7 | 148 | 1.39E-03 | 1.80E-06 | -3.72E-01 | 4.67E-02 | 0.00E+00 | 3.12E-03 | 3.46E-01 | 0.00E+00 | 2.33E-01 |  |
| KIR2DL2 | P43627 | 311 | 3.27E-04 | 0.00E+00 | 2.44E-01 | 2.75E-02 | 0.00E+00 | 0.00E+00 | 2.63E-01 | 0.00E+00 | <0.0025 | 6.35E-01 |
| TFF3 | Q07654 | 136 | 6.71E-03 | 2.14E-05 | -3.67E-01 | 5.28E-02 | 0.00E+00 | 5.44E-03 | 2.44E-01 | 0.00E+00 | <0.0025 | 9.90E-01 |
| LRPAP1 | P30533 | 235 | 0.00E+00 | 0.00E+00 | -2.61E-01 | 2.73E-02 | 0.00E+00 | 0.00E+00 | 2.28E-01 | 0.00E+00 | <0.0025 | 9.88E-01 |
| C2 | P06681 | 151 | 4.55E-02 | 4.50E-05 | 3.06E-01 | 4.86E-02 | 0.00E+00 | 4.11E-03 | 1.49E-01 | 0.00E+00 | <0.0025 | 3.20E-01 |
| CD1C | P29017 | 198 | 2.40E-06 | 0.00E+00 | 5.62E-01 | 5.01E-02 | 0.00E+00 | 1.50E-06 | 9.33E-01 | 0.00E+00 | <0.0025 | 7.80E-01 |
| PLA2G10 | O15496 | 180 | 3.23E-04 | 2.37E-05 | -2.83E-01 | 5.48E-02 | 2.00E-07 | 1.35E-04 | 2.49E-01 | 4.34E-04 | <0.0025 | 7.10E-01 |
| ARHGEF5 | Q12774 | 68 | 2.50E-02 | 2.40E-03 | 2.47E-01 | 5.02E-02 | 8.00E-07 | 7.15E-03 | 7.88E-01 | 1.74E-03 | 9.65E-01 |  |
| GBP4 | Q96PP9 | 107 | 3.14E-03 | 5.80E-03 | 1.77E-01 | 3.63E-02 | 1.20E-06 | 3.17E-03 | 9.18E-01 | 2.60E-03 | 1.58E-01 |  |
| CFB | P00751 | 193 | 4.66E-02 | 3.21E-04 | -1.84E-01 | 3.86E-02 | 1.80E-06 | 2.63E-02 | 4.74E-01 | 3.90E-03 | <0.0025 | 9.18E-01 |
| OPLAH | O14841 | 26 | 1.09E-02 | 5.56E-05 | 6.15E-01 | 1.31E-01 | 2.70E-06 | 6.44E-04 | 6.20E-01 | 5.86E-03 | 8.45E-01 |  |
| TREM2 | Q9NZC2 | 101 | 2.06E-02 | 4.91E-03 | -2.52E-01 | 5.58E-02 | 6.60E-06 | 2.95E-03 | 5.31E-01 | 1.43E-02 | 9.60E-01 |  |
| PLA2G7 | Q13093 | 53 | 3.29E-02 | 4.84E-03 | -3.37E-01 | 7.53E-02 | 7.70E-06 | 3.05E-03 | 6.04E-01 | 1.67E-02 | 3.43E-01 |  |
| FUCA1 | P04066 | 213 | 4.36E-03 | 8.78E-03 | 1.12E-01 | 2.58E-02 | 1.39E-05 | 1.75E-03 | 7.43E-01 | 3.01E-02 | 5.00E-02 |  |
| CTSB | P07858 | 344 | 5.29E-03 | 2.16E-04 | -1.32E-01 | 3.09E-02 | 2.07E-05 | 1.03E-02 | 3.36E-01 | 4.49E-02 | 4.50E-02 | 9.30E-01 |
| **Mean diffusivity** | | | | | | | | | | | | |
| SGSH | P51688 | 302 | 5.00E-07 | 7.42E-03 | 1.98E-01 | 2.94E-02 | 0.00E+00 | 8.22E-03 | 5.62E-01 | 0.00E+00 | <0.0025 | 8.83E-01 |
| ENPP6 | Q6UWR7 | 148 | 1.39E-03 | 2.10E-06 | 3.91E-01 | 6.33E-02 | 0.00E+00 | 1.45E-03 | 8.89E-01 | 0.00E+00 | <0.0025 | 9.40E-01 |
| TFF3 | Q07654 | 137 | 4.19E-02 | 3.04E-04 | 3.46E-01 | 6.27E-02 | 0.00E+00 | 2.33E-02 | 2.87E-01 | 0.00E+00 | <0.0025 | 8.33E-01 |
| APOE | P02649 | 100 | 5.75E-05 | 4.92E-03 | -2.38E-01 | 3.87E-02 | 0.00E+00 | 1.55E-02 | 7.09E-01 | 0.00E+00 | 4.90E-01 |  |
| LRPAP1 | P30533 | 235 | 0.00E+00 | 0.00E+00 | 3.28E-01 | 3.18E-02 | 0.00E+00 | 0.00E+00 | 3.35E-01 | 0.00E+00 | <0.0025 | 7.33E-01 |
| LRRC37A2 | A6NM11 | 218 | 0.00E+00 | 0.00E+00 | 2.38E-01 | 1.95E-02 | 0.00E+00 | 0.00E+00 | 4.20E-01 | 0.00E+00 | 2.50E-03 | 8.08E-01 |
| IL1A | P01583 | 24 | 4.94E-04 | 0.00E+00 | -1.28E+00 | 1.36E-01 | 0.00E+00 | 1.25E-04 | 4.33E-01 | 0.00E+00 | 9.18E-01 |  |
| C2 | P06681 | 151 | 5.95E-03 | 0.00E+00 | -4.81E-01 | 5.65E-02 | 0.00E+00 | 5.60E-06 | 5.85E-02 | 0.00E+00 | <0.0025 | 7.15E-01 |
| BTN2A1 | Q7KYR7 | 208 | 1.00E-07 | 0.00E+00 | -6.81E-01 | 4.56E-02 | 0.00E+00 | 0.00E+00 | 3.84E-01 | 0.00E+00 | <0.0025 | 9.63E-01 |
| GYS1 | P13807 | 205 | 3.12E-03 | 3.00E-07 | 4.50E-01 | 4.33E-02 | 0.00E+00 | 1.31E-03 | 5.29E-01 | 0.00E+00 | 6.83E-01 |  |
| FUCA1 | P04066 | 213 | 8.04E-04 | 1.04E-02 | -1.39E-01 | 2.46E-02 | 0.00E+00 | 5.30E-04 | 3.55E-01 | 0.00E+00 | 4.28E-01 |  |
| CD1C | P29017 | 198 | 1.80E-06 | 0.00E+00 | -7.94E-01 | 5.92E-02 | 0.00E+00 | 1.00E-07 | 2.72E-01 | 0.00E+00 | <0.0025 | 8.30E-01 |
| HLA-DRA | P01903 | 215 | 1.50E-02 | 3.00E-07 | -1.44E-01 | 2.61E-02 | 0.00E+00 | 1.80E-06 | 2.46E-01 | 0.00E+00 | <0.0025 | 6.18E-01 |
| LRP1 | Q07954 | 219 | 0.00E+00 | 0.00E+00 | 3.96E-01 | 3.77E-02 | 0.00E+00 | 0.00E+00 | 2.40E-01 | 0.00E+00 | <0.0025 | 5.23E-01 |
| GZMA | P12544 | 156 | 6.62E-05 | 0.00E+00 | 7.35E-01 | 7.05E-02 | 0.00E+00 | 0.00E+00 | 8.85E-01 | 0.00E+00 | <0.0025 | 9.70E-01 |
| GAST | P01350 | 43 | 4.24E-02 | 0.00E+00 | 1.15E+00 | 1.69E-01 | 0.00E+00 | 4.14E-04 | 9.32E-01 | 0.00E+00 | <0.0025 | 7.08E-01 |
| GBP4 | Q96PP9 | 107 | 3.78E-04 | 1.63E-04 | -1.78E-01 | 3.48E-02 | 3.00E-07 | 4.66E-03 | 4.79E-01 | 6.51E-04 | 9.05E-01 |  |
| PILRB | Q9UKJ0 | 186 | 2.03E-02 | 4.65E-03 | -1.11E-01 | 2.32E-02 | 1.60E-06 | 7.19E-05 | 2.94E-01 | 3.47E-03 | 2.75E-02 | 9.58E-01 |
| PILRA | Q9UKJ1 | 176 | 2.54E-03 | 4.57E-03 | -1.19E-01 | 2.54E-02 | 2.70E-06 | 3.89E-04 | 8.74E-01 | 5.86E-03 | 4.00E-02 | 9.18E-01 |
| PLXDC2 | Q6UX71 | 120 | 3.12E-02 | 3.42E-03 | 3.13E-01 | 6.77E-02 | 3.80E-06 | 2.97E-03 | 8.16E-01 | 8.24E-03 | <0.0025 | 8.65E-01 |
| CD109 | Q6YHK3 | 158 | 7.76E-03 | 7.35E-03 | -1.51E-01 | 3.42E-02 | 9.80E-06 | 3.92E-03 | 5.79E-01 | 2.13E-02 | 2.00E-02 | 9.98E-01 |
| VMO1 | Q7Z5L0 | 142 | 6.90E-03 | 7.76E-03 | 1.40E-01 | 3.20E-02 | 1.19E-05 | 7.06E-04 | 7.97E-01 | 2.58E-02 | 5.58E-01 |  |

**Footnote for supplementary table 6**:

Bonf – corrected IVW p-values; IVW – Inverse variance weighted; MR_P – MR_PRESSO; Nsnp – number of SNPs; SE – standard error; Wmedia – Weighted median; Wmode – Weighted mode; TP53-binding protein 1: TP53BP1; Procollagen C-endopeptidase enhancer 1: PCOLCE; Killer cell immunoglobulin-like receptor 2DL3: KIR2DL3; Butyrophilin subfamily 2 member A1: BTN2A1; Leucine-rich repeat-containing protein 37A2: LRRC37A2; Butyrophilin subfamily 3 member A2: BTN3A2; T-cell surface glycoprotein CD1c: CD1C; Granzyme A: GZMA; Synaptosomal-associated protein 25: SNAP25; Probable serine carboxypeptidase: CPVL; Protein disulfide-isomerase A5: PDIA5; Malignant T-cell-amplified sequence 1: MCTS1; Killer cell immunoglobulin-like receptor 2DL2: KIR2DL2; Protein MENT: MENT; HLA class I histocompatibility antigen, alpha chain E: HLA-E; Epithelial discoidin domain-containing receptor 1: DDR1; Apolipoprotein E: APOE; Transmembrane protein 25: TMEM25; Asporin: ASPN; Tectonic-3: TCTN3; Protogenin: PRTG; Lithostathine-1-beta: REG1B; Scavenger receptor class F member 2: SCARF2; Alpha-2-macroglobulin receptor-associated protein: LRPAP1; Catechol O-methyltransferase: COMT; Dickkopf-like protein 1: DKKL1; Leucine-rich repeat neuronal protein 1: LRRN1; Leukocyte immunoglobulin-like receptor subfamily B member 1: LILRB1; Hyaluronidase-1: HYAL1; Latent-transforming growth factor beta-binding protein 3: LTBP3; Methenyltetrahydrofolate synthase domain-containing protein: MTHFSD; Prostaglandin reductase 1: PTGR1; Tumor necrosis factor ligand superfamily member 12: TNFSF12; Repulsive guidance molecule B: RGMB; Histidine-rich glycoprotein: HRG; Brevican core protein: BCAN; Prolow-density lipoprotein receptor-related protein 1: LRP1; Interleukin-1 alpha: IL1A; Guanylate-binding protein 2: GBP2; Uroporphyrinogen decarboxylase: UROD; Glycerophosphocholine cholinephosphodiesterase ENPP6: ENPP6; Trefoil factor 3: TFF3; Complement C2: C2; Group 10 secretory phospholipase A2: PLA2G10; Rho guanine nucleotide exchange factor 5: ARHGEF5; Guanylate-binding protein 4: GBP4; Complement factor B: CFB; 5-oxoprolinase: OPLAH; Triggering receptor expressed on myeloid cells 2: TREM2; Platelet-activating factor acetylhydrolase: PLA2G7; Tissue alpha-L-fucosidase: FUCA1; Cathepsin B: CTSB; N-sulphoglucosamine sulphohydrolase: SGSH; Glycogen [starch] synthase, muscle: GYS1; HLA class II histocompatibility antigen, DR alpha chain: HLA-DRA; Gastrin: GAST; Paired immunoglobulin-like type 2 receptor beta: PILRB; Paired immunoglobulin-like type 2 receptor alpha: PILRA; Plexin domain-containing protein 2: PLXDC2; CD109 antigen: CD109; Vitelline membrane outer layer protein 1 homolog: VMO1.

# Supplementary Figure 1: Scatter plot showing the pattern of significance in the relationship between proteins and various diseases as well as MRI biomarkers

**
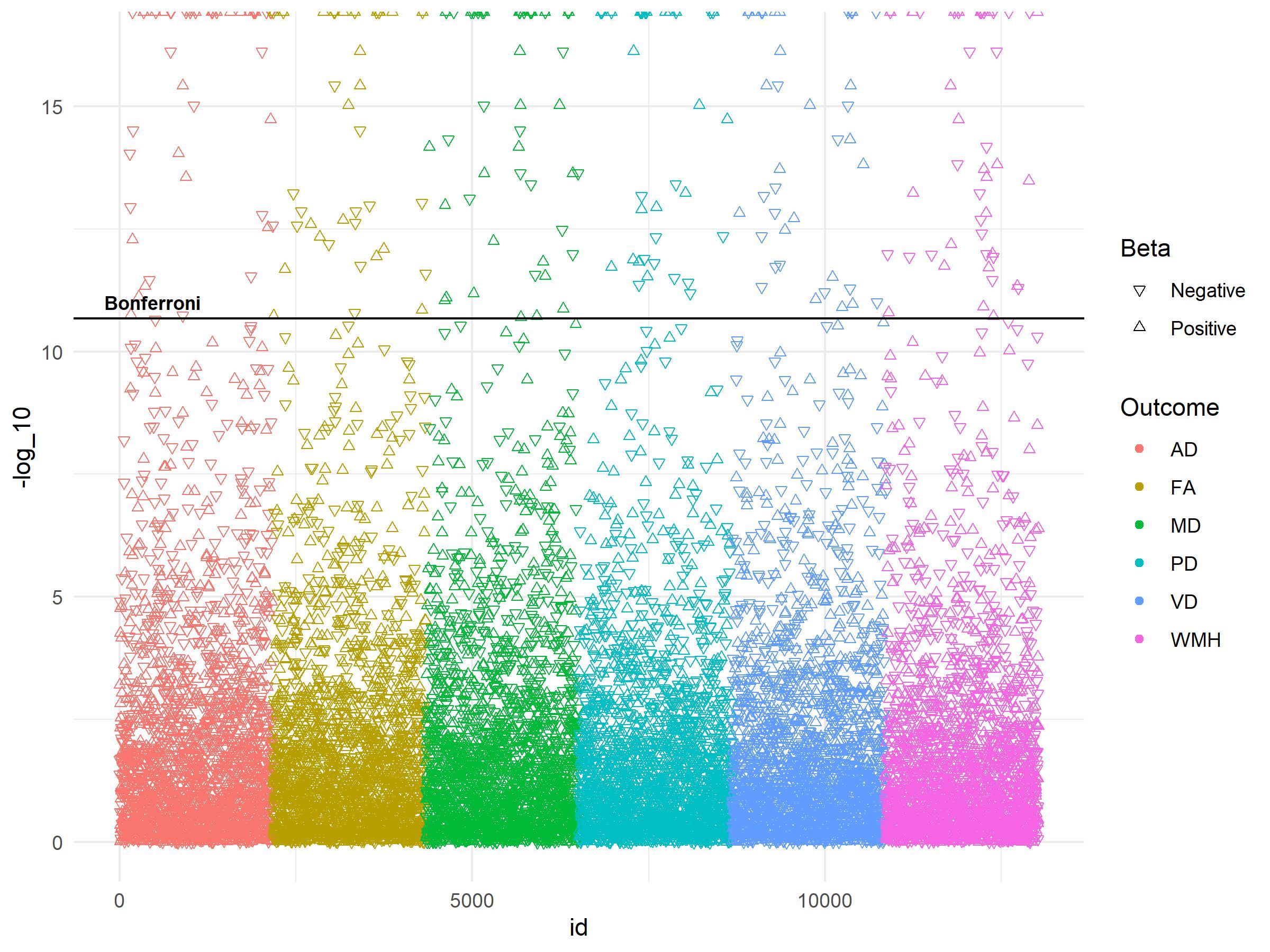
**

**Footnote for Supplementary Figure 1**:

Scatter plot illustrating the significance patterns in the associations between circulating protein levels and various neurological diseases and MRI biomarkers. The vertical axis represents the negative log-transformed p-values (-log10), indicating the strength of the associations. The horizontal axis represents unique identifier indices (id) for proteins analyzed. Different outcomes are color-coded: Alzheimer’s disease (red), fractional anisotropy (brown), mean diffusivity (green), Parkinson’s disease (blue), vascular dementia (cyan), and white matter hyperintensities (magenta). Triangles indicate the direction of the effect, with upward-pointing triangles for positive associations and downward-pointing triangles for negative associations. The horizontal black line marks the threshold for statistical significance.

# References

[1] Kurki MI, Karjalainen J, Palta P, Sipilä TP, Kristiansson K, Donner KM, et al. FinnGen provides genetic insights from a well-phenotyped isolated population. Nature. 2023;613(7944):508-18.

[2] Persyn E, Hanscombe KB, Howson JMM, Lewis CM, Traylor M, Markus HS. Genome-wide association study of MRI markers of cerebral small vessel disease in 42,310 participants. Nat Commun. 2020;11(1):2175.
