# Supplementary material for: Genetic associations of plasma proteomics with dementia subtypes and neuroimaging markers
Source: Alzheimers Dement (Amst). 2025 Oct 21;17(4):e70202. doi: 10.1002/dad2.70202 (PMC12538646; doi:10.1002/dad2.70202)
Supplement: Supplementary file 3 — Supporting Information [file DAD2-17-e70202-s002.docx]

**STROBE-MR checklist of recommended items to address in reports of Mendelian randomization studies**^1^ ^2^

| **Item No.** | **Section** | **Checklist item** | **Page No.** | **Relevant text from manuscript** |
| --- | --- | --- | --- | --- |
| 1 | **TITLE and ABSTRACT** | Indicate Mendelian randomization (MR) as the study’s design in the title and/or the abstract if that is a main purpose of the study | 1 | Genetic associations of plasma proteomics with dementia subtypes and neuroimaging markers  Abstract:  “Multiple potential causal protein-outcome relationships were identified, corroborating known associations and uncovering novel proteins potentially involved in dementia disease processes.” |
|  | **INTRODUCTION** |  |  |  |
| 2 | **Background** | Explain the scientific background and rationale for the reported study. What is the exposure? Is a potential causal relationship between exposure and outcome plausible? Justify why MR is a helpful method to address the study question | 3 | “Previous MR studies have demonstrated individual protein-dementia relationships, such as links between beta-amyloid and tau pathology proteins with Alzheimer's disease ^9^. The availability of large-scale proteomics and genetic datasets enables implementation of high throughput screening methods to identify novel mechanistic pathways, disease biomarkers, and therapeutic targets ^10^.” |
| 3 | **Objectives** | State specific objectives clearly, including pre-specified causal hypotheses (if any). State that MR is a method that, under specific assumptions, intends to estimate causal effects | 3 | “This study examines genetic associations of an extensive panel of plasma proteomics with i) dementia subtypes and ii) neuroimaging markers. The work represents the most comprehensive evaluation of the genetic associations between proteomic markers and dementia ever performed, providing new insights into mechanistic pathways and potential therapeutic targets.” |
|  | **METHODS** |  |  |  |
| 4 | **Study design and data sources** | Present key elements of the study design early in the article. Consider including a table listing sources of data for all phases of the study. For each data source contributing to the analysis, describe the following: |  | Supplementary Table 1 and 2 |
|  | a) | Setting: Describe the study design and the underlying population, if possible. Describe the setting, locations, and relevant dates, including periods of recruitment, exposure, follow-up, and data collection, when available. | 4 | This is a two-sample Mendelian Randomization (MR) study evaluating the genetic associations between plasma proteomics and dementia subtypes as well as neuroimaging markers.  The study leveraged data from 54,219 participants in the UK Biobank for proteomics (exposure) and 429,209 individuals from FinnGen for dementia subtypes (outcomes). Neuroimaging markers were obtained from UK Biobank imaging datasets.  UK Biobank participants were recruited between 2006 and 2010, and follow-up data were obtained from linked electronic health records. FinnGen includes genetic and health record data collected from 2017 onwards from Finnish health registries. Neuroimaging markers were analyzed from the UK Biobank imaging cohort, with GWAS summary statistics published by Persyn et al.  This study used publicly available GWAS summary statistics, primarily based on datasets published between 2020 and 2023. |
|  | b) | Participants: Give the eligibility criteria, and the sources and methods of selection of participants. Report the sample size, and whether any power or sample size calculations were carried out prior to the main analysis | 5 | The study used publicly available genome-wide association study (GWAS) summary statistics from large-scale biobank datasets. The exposure data were derived from the largest available plasma proteomics GWAS conducted in 54,219 UK Biobank participants by Sun et al. The outcome data for dementia subtypes were obtained from the FinnGen cohort, which includes 429,209 individuals of Finnish ancestry, with case definitions based on International Classification of Diseases (ICD) codes recorded in national health registries. The neuroimaging outcome data were sourced from the UK Biobank GWAS on white matter hyperintensities, fractional anisotropy, and mean diffusivity, as published by Persyn et al. |
|  | c) | Describe measurement, quality control and selection of genetic variants | 5 | To select instrumental variables for Mendelian Randomization analysis, single-nucleotide polymorphisms (SNPs) were included if they met a genome-wide significance threshold of p < 5 × 10⁻⁸. Linkage disequilibrium (LD) pruning was performed to ensure the independence of selected SNPs, using an r² threshold of 0.1 and a window size of 10,000 base pairs. This step reduced redundancy among correlated variants.  Harmonization was conducted to align effect alleles between exposure and outcome datasets, ensuring consistency in genetic variant interpretation. Variants were removed if they exhibited strand ambiguity or inconsistent allele frequency distributions between datasets.  For sensitivity analyses, the presence of horizontal pleiotropy was assessed using MR-Egger intercept and Mendelian Randomization-Pleiotropy RESidual Sum and Outlier (MR-PRESSO) methods. Variants flagged as outliers by MR-PRESSO were excluded from the final analyses to minimize potential pleiotropic bias. |
|  | d) | For each exposure, outcome, and other relevant variables, describe methods of assessment and diagnostic criteria for diseases | 4-5 | Plasma protein levels were assessed using genome-wide association study (GWAS) summary statistics from Sun et al., which included 2,923 plasma proteins measured in 54,219 UK Biobank participants. The proteomic data were derived from the Olink platform, which employs Proximity Extension Assay technology. Genetic associations with plasma protein levels were identified using both cis- and trans-protein quantitative trait loci (pQTLs).  Dementia subtypes, including Alzheimer’s disease, vascular dementia, and Parkinson’s disease dementia, were defined based on diagnostic codes from the International Classification of Diseases (ICD) recorded in the FinnGen health registry. The case definitions were as follows: Alzheimer’s disease (ICD-10: G30), vascular dementia (ICD-10: F01), and Parkinson’s disease dementia (ICD-10: F02.3). The FinnGen cohort included 12,348 cases of Alzheimer’s disease, 2,667 cases of vascular dementia, and 589 cases of Parkinson’s disease dementia, with the remaining participants serving as controls.  Neuroimaging markers were obtained from UK Biobank GWAS datasets published by Persyn et al. These included global white matter hyperintensities (WMH), fractional anisotropy (FA), and mean diffusivity (MD). WMH volumes were derived from T1-weighted and T2 FLAIR images. FA and MD values were calculated from diffusion tensor imaging and diffusion MRI, with principal component analysis applied to standardize values across white matter tracts. |
|  | e) | Provide details of ethics committee approval and participant informed consent, if relevant | 6 | This study utilized publicly available genome-wide association study (GWAS) summary statistics, and as such, ethical approval was not required for the Mendelian Randomization analysis. However, the data sources used in this study obtained appropriate ethical approvals and participant informed consent as part of their original research protocols. |
| 5 | **Assumptions** | Explicitly state the three core IV assumptions for the main analysis (relevance, independence and exclusion restriction) as well assumptions for any additional or sensitivity analysis | 4-6 | **Relevance assumption:** The selected genetic variants (single-nucleotide polymorphisms, SNPs) must be strongly associated with the exposure (plasma protein levels). This assumption was addressed by selecting genome-wide significant SNPs (p < 5 × 10⁻⁸) identified in the largest available GWAS for plasma proteomics (Sun et al.), ensuring that only variants with strong evidence of association with protein levels were included.  **Independence assumption:** The genetic variants must be independent of confounders that influence both the exposure and the outcome. This assumption was supported by the use of large GWAS datasets, where potential confounders such as population stratification and relatedness were accounted for through principal component adjustment and exclusion of related individuals.  **Exclusion restriction assumption:** The genetic variants must influence the outcome (dementia subtypes and neuroimaging markers) only through the exposure (plasma protein levels) and not through alternative pathways. To test this assumption, we performed sensitivity analyses, including MR-Egger regression to detect directional pleiotropy and MR-PRESSO to identify and remove pleiotropic outliers. |
| 6 | **Statistical methods: main analysis** | Describe statistical methods and statistics used | 5-6 |  |
|  | a) | Describe how quantitative variables were handled in the analyses (i.e., scale, units, model) | 4-5 | Plasma protein levels were analyzed on a standard deviation (SD) scale, with effect estimates representing the change in protein concentration per SD increase in genetically predicted values. Dementia subtypes were modeled as binary outcomes, with odds ratios (ORs) per SD increase in genetically predicted protein levels. Neuroimaging markers, including white matter hyperintensities, fractional anisotropy, and mean diffusivity, were analyzed as continuous variables, with beta coefficients indicating changes per SD difference in protein levels. The primary MR analysis used the inverse-variance weighted (IVW) method to estimate causal effects, assuming linear relationships between exposure and outcome. |
|  | b) | Describe how genetic variants were handled in the analyses and, if applicable, how their weights were selected | 4-6 | Genetic variants were selected from the largest available genome-wide association study (GWAS) of plasma proteomics, conducted by Sun et al. in 54,219 UK Biobank participants. Single-nucleotide polymorphisms (SNPs) were included as instrumental variables if they reached genome-wide significance (p < 5 × 10⁻⁸) and were associated with plasma protein levels. To ensure independence among variants, linkage disequilibrium (LD) pruning was applied with an r² threshold of 0.1 and a window size of 10,000 base pairs.  The effect sizes of selected SNPs on protein levels, as reported in the exposure GWAS, were used as instrumental variable weights in the Mendelian Randomization (MR) analysis. The harmonization process ensured that effect alleles were aligned between the exposure and outcome datasets. If an instrumental SNP was not present in the outcome dataset, no proxy SNPs were used, and analyses were conducted only with available overlapping variants.  To assess the strength of the genetic instruments, F-statistics were calculated, with values above 10 considered indicative of sufficiently strong instruments. SNPs that showed evidence of horizontal pleiotropy were identified using MR-PRESSO and were removed if flagged as outliers. |
|  | c) | Describe the MR estimator (e.g. two-stage least squares, Wald ratio) and related statistics. Detail the included covariates and, in case of two-sample MR, whether the same covariate set was used for adjustment in the two samples | 5-6 | The primary MR estimator used in this study was the inverse-variance weighted method, which provides a pooled estimate of the causal effect under the assumption that all genetic instruments are valid and free from pleiotropy.  In addition to the inverse-variance weighted method, complementary MR methods were applied to account for potential pleiotropy. MR-Egger regression was used to detect and correct for directional pleiotropy by allowing for an intercept term that indicates horizontal pleiotropy. The weighted median estimator provided robust causal effect estimates. The weighted mode estimator was included to assess the most common causal effect direction when multiple SNPs were used as instruments. The MR-PRESSO method was used to identify and remove outlier SNPs that may bias the causal estimates due to pleiotropic effects.  This study used a two-sample MR approach, where the exposure, plasma proteomics, and outcomes, dementia subtypes and neuroimaging markers, were derived from independent genome-wide association study datasets. Since the original genome-wide association study analyses had already adjusted for key confounders such as age, sex, and population structure using principal component analysis, no additional covariates were included in the MR analysis. The assumption of consistency across the two samples was verified by ensuring that genetic associations were derived from comparable ancestry-matched European populations. |
|  | d) | Explain how missing data were addressed | 4-5 | For missing data, the analysis was based on publicly available genome-wide association study summary statistics, which do not contain individual-level data. Therefore, the issue of missing phenotypic or genotypic data at the participant level was not relevant. |
|  | e) | If applicable, indicate how multiple testing was addressed | 5-6 | Causal estimates were evaluated across multiple MR methods, including inverse-variance weighted, MR-Egger, weighted median, and weighted mode estimators. Consistency of results across these methods was considered an additional validation step to reduce the likelihood of false-positive findings.  Results were interpreted in the context of prior biological evidence and mechanistic plausibility to ensure that statistically significant associations aligned with known or plausible pathways in dementia and neuroimaging phenotypes. |
| 7 | **Assessment of assumptions** | Describe any methods or prior knowledge used to assess the assumptions or justify their validity | 4-6 | The **relevance assumption** was evaluated by selecting single-nucleotide polymorphisms that reached genome-wide significance for plasma protein levels in the largest available genome-wide association study. F-statistics were calculated for each instrument to confirm that weak instrument bias was unlikely, with values greater than 10 considered indicative of sufficiently strong instruments.  The **independence assumption**, which requires that genetic instruments are not associated with confounders, was indirectly assessed through the original genome-wide association study analyses. Additionally, all genetic variants were selected from ancestry-matched European populations to minimize population stratification biases.  The **exclusion restriction assumption** was tested using MR-Egger regression, which detects directional pleiotropy by assessing whether the intercept term deviates from zero. The MR-PRESSO method was also applied to identify and remove outlier single-nucleotide polymorphisms with pleiotropic effects. A leave-one-out sensitivity analysis was performed to determine whether any individual instrumental variable disproportionately influenced the causal estimates. |
| 8 | **Sensitivity analyses and additional analyses** | Describe any sensitivity analyses or additional analyses performed (e.g. comparison of effect estimates from different approaches, independent replication, bias analytic techniques, validation of instruments, simulations) | 5-6 | Sensitivity analyses were performed to assess the robustness of the causal estimates and to account for potential violations of MR assumptions. Several complementary MR methods were used to evaluate the consistency of effect estimates and detect potential biases. First, MR-Egger regression was used to test for directional pleiotropy by assessing whether the intercept term significantly deviated from zero. A nonzero intercept would indicate that the genetic instruments have pleiotropic effects unrelated to the exposure. The weighted mode estimator was also used to detect causal effects when the majority of the valid instruments share a consistent effect.  The MR-PRESSO method was implemented to identify and remove outlier single-nucleotide polymorphisms that exhibited pleiotropic effects, ensuring that causal inferences were not biased by instruments with horizontal pleiotropy. Leave-one-out analysis was conducted to examine whether any single instrumental variable had a disproportionate influence on the results. If the causal estimate changed substantially when an individual variant was removed, this suggested potential bias from that particular instrument. |
| 9 | **Software and pre-registration** |  |  |  |
|  | a) | Name statistical software and package(s), including version and settings used | 6 | The statistical analyses were conducted using **R version** and **TwoSampleMR package**, which was used for Mendelian Randomization analyses, including inverse-variance weighted, Mendelian Randomization-Egger, weighted median, and weighted mode estimators. The **MR-PRESSO package** was applied to detect and correct for pleiotropic outliers. Additional data processing and visualization were performed using the **tidyverse** and **ggplot2** packages. Linkage disequilibrium clumping was conducted using the **PLINK software** to ensure the selection of independent genetic instruments. |
|  | b) | State whether the study protocol and details were pre-registered (as well as when and where) |  | The study protocol was **not pre-registered** in an official trial registry, as it is a MR study using publicly available summary statistics. However, all data sources, including genome-wide association study datasets and their references, were documented in the manuscript and supplementary material. |
|  | **RESULTS** |  |  |  |
| 10 | **Descriptive data** |  |  |  |
|  | a) | Report the numbers of individuals at each stage of included studies and reasons for exclusion. Consider use of a flow diagram | 7-8 | The **exposure dataset** for plasma proteomics included **54,219 individuals** from the **UK Biobank**, based on the genome-wide association study conducted by **Sun et al.**  The **outcome dataset** for dementia subtypes was derived from **FinnGen**, with a total of **429,209 participants**, including **12,348 cases of Alzheimer’s disease, 2,667 cases of vascular dementia,** and **589 cases of Parkinson’s disease dementia**, with the remaining participants serving as controls.  The **outcome dataset** for neuroimaging markers was obtained from **UK Biobank**, with sample sizes varying across traits. White matter hyperintensities were analyzed in **42,310 individuals**, while fractional anisotropy and mean diffusivity were assessed in approximately **17,000 individuals** each, based on genome-wide association studies published by **Persyn et al.** |
|  | b) | Report summary statistics for phenotypic exposure(s), outcome(s), and other relevant variables (e.g. means, SDs, proportions) | 7-10 | The exposure dataset for plasma proteomics included 54,219 individuals from the UK Biobank. A total of 2,923 plasma proteins were analyzed using genome-wide association study summary statistics from Sun et al. Genetic instruments were selected for proteins that had at least one genome-wide significant single-nucleotide polymorphism with a p-value less than 5 × 10⁻⁸. Effect sizes for protein levels were reported in standard deviation units.  The outcome dataset for dementia subtypes was obtained from FinnGen and included 429,209 participants. The case-control breakdown was as follows: Alzheimer’s disease had 12,348 cases and 416,861 controls, vascular dementia had 2,667 cases and 426,542 controls, and Parkinson’s disease dementia had 589 cases and 428,620 controls. Dementia risk was modeled as a binary outcome, with effect estimates reported as log-odds ratios per standard deviation increase in genetically predicted protein levels.  The neuroimaging outcome dataset was derived from the UK Biobank. White matter hyperintensities were analyzed in 42,310 individuals, fractional anisotropy in 17,663 individuals, and mean diffusivity in 17,467 individuals, based on genome-wide association studies published by Persyn et al. Neuroimaging markers were analyzed as continuous variables, with effect estimates reported as beta coefficients per standard deviation difference in genetically predicted protein levels. |
|  | c) | If the data sources include meta-analyses of previous studies, provide the assessments of heterogeneity across these studies |  | This study primarily relied on summary statistics from single large-scale genome-wide association studies rather than meta-analyses of multiple independent cohorts. |
|  | d) | For two-sample MR:  i.  Provide justification of the similarity of the genetic variant-exposure associations between the exposure and outcome samples  ii.  Provide information on the number of individuals who overlap between the exposure and outcome studies | 4-6 | The exposure dataset, which included plasma proteomics data from 54,219 UK Biobank participants, and the outcome datasets, derived from FinnGen (for dementia subtypes) and UK Biobank (for neuroimaging markers), were both based on European-ancestry populations, reducing potential biases due to population stratification.  The genetic variant-exposure associations were derived from the largest available genome-wide association study on plasma proteomics, ensuring strong instrumental variables for MR analysis. The linkage disequilibrium structure and allele frequencies were comparable between the exposure and outcome datasets, supporting the validity of the genetic instruments.  Regarding sample overlap, there was minimal risk of overlap between the exposure and outcome datasets. The plasma proteomics genome-wide association study was conducted exclusively in UK Biobank participants, whereas the dementia outcome dataset was sourced from FinnGen, a completely independent cohort with no individual-level data shared between the two studies. For neuroimaging markers, both the exposure and outcome datasets were based on UK Biobank participants, but the proteomics and imaging genome-wide association studies were conducted as separate analyses, minimizing overlap concerns. The degree of overlap was not explicitly quantified in the genome-wide association study publications; however, since this was a two-sample MR design, any potential sample overlap would be expected to have a minimal impact on the causal estimates. |
| 11 | **Main results** |  |  |  |
|  | a) | Report the associations between genetic variant and exposure, and between genetic variant and outcome, preferably on an interpretable scale | 7-10 | For the genetic variant-exposure associations, effect sizes were obtained from the genome-wide association study conducted by Sun et al., which analyzed 2,923 plasma proteins in 54,219 UK Biobank participants. The associations were expressed in standard deviation units of protein levels per effect allele of the single-nucleotide polymorphisms used as instrumental variables. Only genome-wide significant associations with a p-value less than 5 × 10⁻⁸ were retained, and linkage disequilibrium pruning with an r² threshold of 0.1 was applied to ensure the independence of selected variants. |
|  | b) | Report MR estimates of the relationship between exposure and outcome, and the measures of uncertainty from the MR analysis, on an interpretable scale, such as odds ratio or relative risk per SD difference | 7-10 | For dementia subtypes, including Alzheimer’s disease, vascular dementia, and Parkinson’s disease dementia, the causal effect estimates were expressed as odds ratios per standard deviation increase in genetically predicted plasma protein levels. The inverse-variance weighted method was used as the primary Mendelian Randomization approach, with complementary sensitivity analyses including Mendelian Randomization-Egger, weighted median, and weighted mode estimators. The 95 percent confidence intervals and p-values were reported to indicate the precision and statistical significance of the estimates.  For neuroimaging markers, including white matter hyperintensities, fractional anisotropy, and mean diffusivity, the causal estimates were expressed as beta coefficients per standard deviation increase in genetically predicted plasma protein levels. The same Mendelian Randomization methods were applied, and the results were presented with corresponding confidence intervals and p-values. |
|  | c) | If relevant, consider translating estimates of relative risk into absolute risk for a meaningful time period |  | Since the study used a two-sample Mendelian Randomization approach with genome-wide association study summary statistics, absolute risk estimates were not calculated. |
|  | d) | Consider plots to visualize results (e.g. forest plot, scatterplot of associations between genetic variants and outcome versus between genetic variants and exposure) |  | The analysis included forest plots to illustrate causal effect estimates across different Mendelian Randomization methods and scatter plots to compare associations between genetic variants and exposure versus genetic variants and outcome. Please refer to figure 2 and 3, as well as to supplementary figure 1. |
| 12 | **Assessment of assumptions** |  |  |  |
|  | a) | Report the assessment of the validity of the assumptions | 4-6 | For the relevance assumption, instrumental variables were selected based on genome-wide significance with a p-value threshold of less than 5 × 10⁻⁸ from the largest available genome-wide association study of plasma proteomics. The strength of the genetic instruments was evaluated using F-statistics, with values greater than 10 considered indicative of sufficiently strong instruments.  For the independence assumption, potential confounding due to population stratification and relatedness was addressed by ensuring that genome-wide association study analyses had adjusted for age, sex, and principal components. Additionally, genetic variants were selected from ancestry-matched European populations to minimize confounding due to population structure.  For the exclusion restriction assumption, several sensitivity analyses were performed to detect and account for pleiotropy. Mendelian Randomization-Egger regression was used to test for directional pleiotropy, where a significant nonzero intercept would indicate potential violations. The Mendelian Randomization-PRESSO method was applied to identify and remove outlier single-nucleotide polymorphisms with pleiotropic effects. The weighted median and weighted mode estimators were included to provide robust causal estimates even when some instrumental variables were invalid. |
|  | b) | Report any additional statistics (e.g., assessments of heterogeneity across genetic variants, such as *I^2^*, Q statistic or E-value) | 5-6 | To ensure the validity of the single-nucleotide polymorphisms and address potential heterogeneity and directional pleiotropy, complementary analyses were conducted, including Mendelian Randomization-Egger regression, weighted median, and weighted mode methods. |
| 13 | **Sensitivity analyses and additional analyses** |  |  |  |
|  | a) | Report any sensitivity analyses to assess the robustness of the main results to violations of the assumptions |  | Sensitivity analyses were conducted to assess the robustness of the causal estimates and to test for violations of the core MR assumptions. Several complementary methods were used to ensure the validity of the findings.  MR-Egger regression was performed to detect and correct for directional pleiotropy. A significant nonzero intercept in the MR-Egger analysis would indicate that some instrumental variables influence the outcome through pathways other than the exposure, violating the exclusion restriction assumption.  The weighted median estimator was used as a robustness check, as it provides valid causal estimates even if up to 50 percent of the instrumental variables are invalid. The weighted mode estimator was also applied, which is more robust when the majority of valid instruments share a similar causal effect.  Leave-one-out analyses were performed by sequentially removing individual single-nucleotide polymorphisms to evaluate whether any single instrument disproportionately influenced the causal estimates. If the effect size changed significantly upon removing a particular variant, it could suggest that the single-nucleotide polymorphism had pleiotropic effects or was a weak instrument.  The MR-PRESSO method was applied to detect and correct for outlier single-nucleotide polymorphisms that exhibited evidence of pleiotropy.  The inverse-variance weighted method was used as the primary estimator, but results were compared across different MR methods, including MR-Egger, weighted median, and weighted mode, to assess the consistency of causal estimates. If the causal effect remained stable across methods, this provided additional confidence in the robustness of the findings. |
|  | b) | Report results from other sensitivity analyses or additional analyses | 5-6 | The study focused on assessing the robustness of the MR estimates through complementary MR methods, including MR-Egger, weighted median, weighted mode, leave-one-out analyses, and MR-PRESSO. |
|  | c) | Report any assessment of direction of causal relationship (e.g., bidirectional MR) |  | Bidirectional MR was not conducted in this study. |
|  | d) | When relevant, report and compare with estimates from non-MR analyses |  | Non-MR analyses were not conducted in this study. |
|  | e) | Consider additional plots to visualize results (e.g., leave-one-out analyses) |  | Please refer to figure 2 and 3, as well as to supplementary figure 1 for visualization of results. |
|  | **DISCUSSION** |  |  |  |
| 14 | **Key results** | Summarize key results with reference to study objectives | 11 | The key results were summarized in the discussion section with reference to the study objectives. |
| 15 | **Limitations** | Discuss limitations of the study, taking into account the validity of the IV assumptions, other sources of potential bias, and imprecision. Discuss both direction and magnitude of any potential bias and any efforts to address them | 16-17 | The limitations of the study were discussed, taking into account the validity of the instrumental variable assumptions, potential sources of bias, and imprecision in the estimates. The discussion included considerations of pleiotropy, population stratification, the generalizability of findings, and the constraints of using genome-wide association study summary statistics for MR. Efforts to address these limitations, such as sensitivity analyses and pleiotropy correction methods, were also described. |
| 16 | **Interpretation** |  |  |  |
|  | a) | Meaning: Give a cautious overall interpretation of results in the context of their limitations and in comparison with other studies | 11-17 | The results were interpreted in the discussion, considering the study's limitations and comparing findings with previous research. The interpretation acknowledged the strengths of the MR approach in inferring causality while highlighting potential biases, such as pleiotropy and population stratification. Comparisons with observational and experimental studies were included to contextualize the findings within existing literature. |
|  | b) | Mechanism: Discuss underlying biological mechanisms that could drive a potential causal relationship between the investigated exposure and the outcome, and whether the gene-environment equivalence assumption is reasonable. Use causal language carefully, clarifying that IV estimates may provide causal effects only under certain assumptions | 11-15 | The discussion included an evaluation of the potential **biological mechanisms** linking plasma protein levels to dementia subtypes and neuroimaging markers. Proposed pathways were based on prior literature and mechanistic insights, considering the role of specific proteins in neuroinflammation, vascular health, and neurodegeneration. |
|  | c) | Clinical relevance: Discuss whether the results have clinical or public policy relevance, and to what extent they inform effect sizes of possible interventions | 16 | The discussion addressed the **clinical relevance** of the findings, considering their potential implications for dementia risk stratification, biomarker discovery, and therapeutic targeting. The results were evaluated in the context of **identifying plasma proteins as potential causal contributors to neurodegeneration,** which could inform precision medicine approaches. |
| 17 | **Generalizability** | Discuss the generalizability of the study results (a) to other populations, (b) across other exposure periods/timings, and (c) across other levels of exposure | 11-17 | The discussion addressed the **generalizability** of the study findings by considering several key factors:  a) The study primarily used genetic data from European ancestry cohorts, which may limit the applicability of the findings to populations of different ethnic backgrounds. Differences in genetic architecture, environmental exposures, and baseline dementia risk across populations should be considered when extrapolating these results.  b) The MR approach estimates lifelong genetically predicted effects of plasma proteins, which may not directly correspond to short-term or time-dependent changes in protein levels due to disease progression, aging, or external interventions. The findings provide insights into potential causal pathways but do not capture temporal variations in protein expression.  c) The MR estimates reflect the effect of genetically predicted differences in protein levels rather than pharmacologically or environmentally induced changes. The results may not directly translate to therapeutic interventions, as genetically influenced protein changes may differ in magnitude or mechanism from those induced by drugs or lifestyle modifications. |
|  | **OTHER INFORMATION** |  |  |  |
| 18 | **Funding** | Describe sources of funding and the role of funders in the present study and, if applicable, sources of funding for the databases and original study or studies on which the present study is based | 18 | The funding sources were described in the publication. |
| 19 | **Data and data sharing** | Provide the data used to perform all analyses or report where and how the data can be accessed, and reference these sources in the article. Provide the statistical code needed to reproduce the results in the article, or report whether the code is publicly accessible and if so, where | 18 | The data availability were described in the publication. |
| 20 | **Conflicts of Interest** | All authors should declare all potential conflicts of interest | 18 | The conflicts of interest were described in the publication. |

This checklist is copyrighted by the Equator Network under the Creative Commons Attribution 3.0 Unported (CC BY 3.0) license.

1. Skrivankova VW, Richmond RC, Woolf BAR, Yarmolinsky J, Davies NM, Swanson SA, et al. Strengthening the Reporting of Observational Studies in Epidemiology using Mendelian Randomization (STROBE-MR) Statement. JAMA. 2021;under review.

2. Skrivankova VW, Richmond RC, Woolf BAR, Davies NM, Swanson SA, VanderWeele TJ, et al. Strengthening the Reporting of Observational Studies in Epidemiology using Mendelian Randomisation (STROBE-MR): Explanation and Elaboration. BMJ. 2021;375:n2233.
